# Characterisation of negative allosteric modulators of the calcium-sensing receptor, CaSR, for repurposing as a treatment for asthma

**DOI:** 10.1124/jpet.120.000281

**Published:** 2020-10-28

**Authors:** Polina L. Yarova, Ping Huang, Martin W. Schepelmann, Richard Bruce, Rupert Ecker, Robert Nica, Vsevolod Telezhkin, Daniela Traini, Larissa Gomes dos Reis, Emma J. Kidd, William R. Ford, Kenneth J. Broadley, Benson M. Kariuki, Christopher J. Corrigan, Jeremy P.T Ward, Paul J. Kemp, Daniela Riccardi

**Affiliations:** School of Biosciences, Cardiff University, Cardiff, UK; School of Biosciences, Cardiff University, Cardiff, UK; Institute for Pathophysiology and Allergy Research, Medical University of Vienna, Vienna, Austria; School of Biosciences, Cardiff University, Cardiff, UK; TissueGnostics GmbH, Vienna, Austria; TissueGnostics GmbH, Vienna, Austria; School of Dental Sciences, University of Newcastle, UK; Woolcock Institute of Medical Research, The University of Sydney, Sydney, Australia; School of Pharmacy, Cardiff University, Cardiff, UK; School of Chemistry, Cardiff University, Cardiff, UK; School of Immunology & Microbial Sciences, King’s College London, London, UK; School of Biosciences, Cardiff University, Cardiff, UK

## Abstract

Asthma is still an incurable disease and there is a recognised need for novel small molecule therapies for people with asthma, especially those poorly controlled by current treatments. We previously demonstrated that calcium-sensing receptor negative allosteric modulators (CaSR NAMs), calcilytics, uniquely suppress both airway hyperresponsiveness (AHR) and inflammation in human cells and murine asthma surrogates. Here we assess the feasibility of repurposing four CaSR NAMs, originally developed for oral therapy for osteoporosis and previously tested in the clinic, as a novel, single and comprehensive topical anti-asthma therapy. We address the hypotheses, using murine asthma surrogates, that topically-delivered CaSR NAMs (i) abolish AHR; (ii) are unlikely to cause unwanted systemic effects; (iii) are suitable for topical application; (iv) inhibit airways inflammation to the same degree as the current standard of care, inhaled corticosteroid (ICS), and furthermore inhibit airways remodelling. All four CaSR NAMs inhibited poly-L-arginine-induced AHR in naïve mice, and suppressed both AHR and airways inflammation in a murine surrogate of acute asthma, confirming class specificity. Repeated exposure to inhaled CaSR NAMs did not alter blood pressure, heart rate or serum calcium concentrations. Optimal candidates for repurposing were identified based on anti-AHR/inflammatory activities, PK/PD, formulation and micronization studies. Whereas both inhaled CaSR NAMs and inhaled corticosteroids reduced airways inflammation, only the former prevented goblet cell hyperplasia in a chronic asthma model. We conclude that inhaled CaSR NAMs are likely a single, safe and effective topical therapy for human asthma, abolishing AHR, suppressing airways inflammation and abrogating some features of airways remodelling.

## Introduction

More than 300 million people suffer from asthma worldwide, with a substantial burden of morbidity and mortality. The current mainstay therapies of inhaled corticosteroids (ICS) and bronchodilators control symptoms resulting from airways obstruction reasonably well in most patients, but may not be as effective at altering the natural history of airways obstruction and remodelling (An et al., 2007; O’Byrne et al., 2019). As a consequence, severe, therapy-resistant obstruction develops in a significant minority of patients, resulting in life-long impairment of quality of life, increased risk of hospital admission and death (Hansbro et al., 2017). Furthermore, no current anti-asthma drug *directly* targets bronchial smooth muscle hyperresponsiveness, a critical contributor to airways obstruction and the fundamental physiological abnormality characterising asthma (Sterk and Bel, 1989; An et al., 2007). Therapies based on monoclonal antibodies (biologicals) targeting IgE binding or asthma-related cytokines, reduce obstruction and exacerbations to an unpredictable extent in some, but not all patients, and are further limited by administration logistics and cost (Walsh, 2017). Consequently, there is a strong need for the development of novel small molecule drugs, preferably delivered topically using commercially available devices, targeting both bronchial smooth muscle hyperresponsiveness and airways inflammation.

The calcium-sensing receptor (CaSR) is a G-protein coupled receptor originally identified as the master controller for serum ionised calcium levels (Brown et al., 1993). It is a multimodal receptor activated not only by divalent cations (Ca^2+^, Mg^2+^), but also by polycations released by cells involved in asthmatic airways inflammation such as spermine, spermidine and eosinophil-derived cationic proteins (Kurosawa et al., 1992; Maarsingh et al., 2008; Yarova et al., 2015). The CaSR is expressed in multiple tissues, including airways smooth muscle and inflammatory cells, and has been implicated in numerous cellular activities and several disorders independently of calcium homeostasis (Riccardi and Kemp, 2012; Yarova et al., 2015; Brennan et al., 2016; Hannan et al., 2018). Notably, the CaSR is an important regulator of inflammation via activation of the NLRP3 inflammasome (Lee et al., 2012; Rossol et al., 2012). Using murine asthma surrogates and cultured human asthmatic smooth muscle cells, we have recently presented evidence consistent with the hypothesis that AHR in human asthma reflects abnormal expression of the CaSR, and shown that small molecule CaSR negative allosteric modulators (NAMs), also known as calcilytics, abolish both AHR and airways inflammation (Yarova et al., 2015; Corrigan, 2020).

We have previously used commercially available CaSR NAMs as pharmacological tools, but their limited bioavailability detracts from their suitability for clinical development (Yarova et al., 2015). However, several pharmaceutical companies have developed and assessed (up to Phase 2 clinical trials) systemically delivered CaSR NAMs for efficacy in reversing osteoporosis, namely the amino alcohols NPSP-795, Ronacaleret and JTT-305, and the quinazolin-2-one AXT-914 (Kumar et al., 2010; Caltabiano et al., 2013; Halse et al., 2014; John et al., 2014). Although exhibiting good safety and tolerability profiles, they were found not to be efficacious for post-menopausal osteoporosis and induced hypercalcaemia in some patients; their further development for treating osteoporosis was therefore halted (Nemeth et al., 2018). As CaSR NAMs have the potential advantage of suppressing both AHR and inflammation, in this investigation we address the hypothesis that these four clinically tested CaSR NAMs can be repurposed as a novel, single drug, topical anti-asthma therapy which will obviate the need for both topical bronchodilator and corticosteroid therapy. To achieve this we aimed to characterise, in terms of pharmacology, safety, efficacy, PK/PD, and lack of systemic effects, the suitability of existing calcilytics for topical therapy of human asthma, and to compare their effects with those of the current standard of care, topical ICS, in a chronic murine model of Th2/IgE-driven asthma.

## Methods

### In vitro studies

#### Determination of CaSR NAM efficacies and potencies in vitro

All chemicals and reagents were obtained from Sigma-Aldrich, UK, unless specified. HEK293 cells stably expressing human CaSR (HEK-CaSR) (Ward et al., 2013) were loaded with the Ca^2+^ indicator fura-2 AM and calcium imaging was carried out as previously described (Yarova et al., 2015) using an inverted microscope (Olympus IX71) and fluorescence source (Xenon lamp). Extracellular solution contained (in mM): 135 NaCl, 5 KCl, 5 N-2-hydroxyethylpiperazine-N’-2-ethanesulfonic acid (HEPES), 10 glucose, 1.2 MgCl_2_, 0.5 CaCl_2_, pH=7.4. A rapid perfusion system was used to alter extracellular Ca^2+^ concentration and apply test compounds.

#### Patch clamp recordings of the effects of CaSR NAMs on the L-type Ca^2+^ channels

Some CaSR NAMs are structural derivatives of dihydropyridines, compounds that affect the current via L-type Ca^2+^ channel. To examine whether CaSR NAMs had any effect on these channels, voltage and current recordings were made using conventional patch-clamp in the whole-cell configuration in HEK293 cells stably expressing the α1c subunit of L-type Ca^2+^ channels (CaV1.2) (Fearon et al., 2000) employing an Axopatch 200B amplifier interfaced to a computer running pClamp 9 using a Digidata 1322A A/D interface (Molecular Devices, Sunnyvale, CA, U.S.A.). Recordings were digitized at 10 kHz and low-pass filtered at 2 or 5 kHz using an 8-pole Bessel filter. The bath solution for measurements of Ca^2+^ current contained (in mM): 95 NaCl, 5 CsCl, 0.6 MgCl_2_, 20 BaCl_2_, 10 HEPES, 15 D-Glucose D-glucose, 20 Tetraethylammonium chloride (TEA), pH was adjusted to 7.4 by 1M NaOH. The standard pipette solution for Ca^2+^ current contained (in mM): 120 CsCl, 20 TEA, 2 MgCl_2_, 10 ethylene-glycol-tetra-acetic acid (EGTA); 10 HEPES, 2 Na_2_-ATP, 1.6 Na-GTP, pH was adjusted to 7.2 with CsOH. For episodic stimulation the following voltage-clamp protocol was used: holding potential -70 mV and 450 ms steps to 0 mV with 5 s time interval. Cell capacitance and series resistance were measured and compensated at between 60 and 90%. Tested compounds were introduced through the continuous flow system. Pipette resistances were 8-10 MΩ when filled with the pipette solutions. Data were analysed using Clampfit 10.2.

### Animal studies

#### Animal procedures

All animal procedures conformed to the regulations of the Animals (Scientific Procedures) Act 1986 and Declaration of Helsinki conventions for the use and care of animals, and were approved by the Home Office (UK) and local ethics committee. Two to three month old BALB/c mice were obtained from Envigo (UK), maintained under standard conditions with food and water *ad libitum*, and used in experiments at 6-8 weeks of age. According to the *a priori* F test power calculations for one-way omnibus ANOVA, with α error probability of 0.05, power 0.8, and effect size 0.7, six mice were needed for each treatment group of the five groups per each experiment (30 animals in total per experiment; G*Power v.1.3.9.6). Male mice were used in all experiments with the exception of the *in vivo* head-to-head comparison of CaSR NAM with ICS. For these experiments only, where inflammation was measured as the primary readout, female mice were used, owing to the heightened inflammatory response compared to male mice (Melgert et al., 2005; Blacquiere et al., 2010). Animals were assigned at random to experimental groups and *in vivo* and *in vitro* data measured and analysed by operators blinded to the experimental conditions.

#### Ex vivo measurements of airway reactivity

Following humane euthanasia by a schedule 1 method (i.p. pentobarbital overdose), tracheas were isolated from the mice and cleaned as previously described (Donovan et al., 2013). Each tracheal ring of 2 mm in length was then mounted into jaws of a small vessel wire myograph (DMT 610, Danish Myo Technology, Denmark) using steel wires, and left to equilibrate at 37°C in 5% CO_2_/95%O_2_-bubbled Krebs buffer containing 2 mM Ca^2+^ to mimic pro-inflammatory conditions (118 mM NaCl, 3.4 mM KCl, 1.2 mM MgSO_4_, 1.2 mM KH_2_PO_4_, 25 mM NaHCO_3_, 2 mM CaCl_2_, 11 mM glucose) for 20 min, after which the tracheal rings were gradually stretched to 5 mN, washed, and left to equilibrate for another 20 min. Parallel experiments were also carried out in Krebs solution containing 1 mM Ca^2+^, to mimic physiological conditions. Tracheas were then tested for viability with high K^+^ (40 mM and 80 mM), and an acetylcholine (ACh) concentration-response curves generated by cumulative addition of ACh to the bath. Preparations were then washed with Krebs buffer before being half-maximally contracted with ACh at its EC_50_ (10-30 nM). Cumulative concentration-response curves were then obtained for the CaSR NAMs and vehicle control (DMSO in Krebs buffer).

#### Drug nebulisation

NPSP-795, Ronacaleret, JTT-305 and AXT-914 were custom-synthesised by Sygnature Discovery (BioCity, Nottingham, UK) while NPS2143 (positive control, (Yarova et al., 2015)) was obtained from Tocris Bioscience (Bristol, UK). Compounds were delivered into a Perspex chamber using a SideStream nebuliser (Philips Respironics, Philips Hospital & Health Care Amsterdam, Noord-Holland, NL) equipped with a compressor (PulmoStar, Sunrise Medical, West Midlands, DY5 2LD UK). Dosage estimation was carried out as described in the Supplementary Materials.

#### Measurements of airway obstruction

Measurements of airways obstruction were carried out using non-invasive barometric plethysmography (Buxco Research Systems, DSI, St Paul, MN, USA) in unrestrained, conscious mice, as previously described (Fernandez-Rodriguez et al., 2010; Yarova et al., 2015), where enhanced pause (Penh) was used as an indicator of airways obstruction to study AHR, as previously described (Hamelmann et al., 1997; Yarova et al., 2015). Briefly, after establishing baseline Penh, a standard nebulised methacholine (MCh) challenge was performed *in situ* (0.1 to 100 mg/ml in saline; 3-min Penh recorded per dose). Increases in Penh in response to MCh inhalations were first recorded in naive animals, 24 hours later the recordings were repeated following the treatment by inhalation of the nebulized compounds, and consequent changes in Penh were expressed as ΔPenh.

#### Polycation-induced AHR

Poly-L-arginine (PLA), a mimetic of eosinophil major basic protein, activates the CaSR and induces AHR in mice following inhalation (Yarova et al., 2015). Mice were exposed to nebulised PLA (6 μM in 0.2% DMSO in PBS), PLA + CaSR NAM, or vehicle for 30 min immediately before MCh challenge (01-100 mg/ml). In separate studies to determine the drug duration of effects (pharmacodynamics, PD), animals were exposed to aerosolised calcilytics or vehicle zero, one, two, four, eight or 24 hours prior to PLA treatment.

#### Determination of the effects of CaSR NAMs in an acute asthma model

Mice were sensitised with ovalbumin from chicken eggs (OVA) on days 0 and 5 using an i.p. injection of OVA (100 μg/mouse) in 10% (50 mg/mouse) aluminium hydroxide in PBS as previously described (Yarova et al., 2015). Fourteen days after the second injection, mice were challenged by inhalation of 0.5% OVA aerosol for one hour twice on the same day, 4 hours apart. CaSR NAMs or corresponding vehicle treatments were administered via nebulisation 1 hour prior to the first, and 4 hours after the final OVA challenge. At the end of the experiments the mice were euthanized humanely as described above and bronchoalveolar lavage fluid (BALF) collected as previously described (Yarova et al., 2015) and analysed for cell numbers using a Luna FL automated cell counter (Labtech, Heathfield, East Sussex, UK). Lung sections were Masson trichrome-stained and quantitative image analysis was carried out using TissueFAXS image analysis software (TissueGnostics, Vienna, Austria).

#### Systemic effects of repeated inhaled calcilytic exposure

As DMSO may accumulate in tissues with repetitive dosing, the vehicle in these experiments was changed to 0.3% propylene glycol in PBS (for NPSP-795, Ronacaleret and JTT-305) or 0.27% propylene glycol + 0.03% ethanol in PBS (for AXT-914). Mice were placed in a Perspex chamber and exposed to nebulised CaSR NAMs or vehicle for one hour daily for five days. Following the final exposure, blood pressure and heart rate were measured as described previously using CODA non-invasive blood pressure measurement system (Kent Scientific, USA) (Schepelmann et al.). At the end of the experiment, the animals were euthanized humanely and BALF collected for determination of inflammatory cellular infiltration. Because the CaSR is the master controller of systemic Ca^2+^ concentrations, blood samples were also collected at the end of the experiments to measure free ionized serum Ca^2+^ concentrations, as an indication of a possible systemic drug overspill following CaSR NAM nebulisation.

#### Pharmacokinetics (PK)

PK studies were carried out by Axis BioServices Ltd. (Coleraine, Londonderry, N.I.) following direct intra-tracheal (IT) instillation of CaSR NAMs into anaesthetised (100 mg/kg ketamine and 10 mg/kg xylazine) BALB/c mice (Penn-Century™ aerosoliser, Heathfield, United Kingdom). Compounds were re-suspended in 3% DMSO, 97% (5% EtOH/5% glucose in water) all at nominal concentrations of 17.5µg/kg, then filtered through a 0.45 μm pore filter and the filtered solution administered to the mice (Table 1). IT cassette were dosed as a solution of 38 µg/kg (NPSP-795, Ronacaleret and AXT-914) or 26 µg/kg (JTT-305). Whole blood (150μl) was collected at each time point using a lateral tail vein bleed into microvettes coated with K_2_EDTA. Plasma was collected by centrifugation at 2,000 g for 5 min. Animals were then humanely killed euthanised, and the lungs and trachea removed and snap-frozen. Samples were analysed by XenoGesis (BioCity, Nottingham, UK), where concentrations of the tested compounds were determined using a ThermoTM TSQ Quantiva and Vanquish UHPLC system (Fisher, United Kingdom).

#### Head-to-head comparison of inhaled CaSR NAM and inhaled corticosteroids (ICS) in a chronic asthma model

Inhaled corticosteroids (ICS) are recommended as a first line of defence for the treatment of persistent asthma in all guidelines (Ye et al., 2017; Chipps et al., 2020). The anti-inflammatory and anti-remodelling effects of the CaSR NAM NPSP-795 were compared with those of inhaled fluticasone phosphate (FP) in a murine surrogate of Th2/IgE-driven asthma. Mice were initially sensitised on days 0 and 10 by i.p. injection of 50 μg OVA and 50 mg aluminium hydroxide in PBS and then challenged every other day from day 21 to day 30 with inhaled 0.5% OVA delivered by aerosol for one hour. From day 25 mice were treated with the aerosolised NPSP-795 (6 μM), FP (0.25 mg) or vehicle (0.01 % Tween-80/0.03% DMSO) delivered twice daily for 15 min, 1 hour prior to and 7 hours after each OVA challenge for six days. At the end of the experiment, the BALF was collected and analysed as described above, while the lungs were infused with normal buffer formalin, formalin-fixed, paraffin-embedded and 5 µm sections cut for Masson’s trichrome and haematoxylin and eosin staining and immunohistochemical analysis.

### Crystallization and phase characterization

Samples of NPSP-795, Ronacaleret and AXT-914 were crystallised after dissolution in acetone followed by slow evaporation of the solvent at room temperature over several days. AXT-914 was also crystallised from ethanol following the same procedure. For structural characterization, single crystal data were recorded on an Agilent SuperNova Dual Atlas diffractometer (Agilent, Santa Clara, CA) equipped with an Oxford Cryosystems cooling apparatus (Oxford Cryosystems, Oxford, UK). Crystal structures were solved and refined using SHELXS and SHELXL (Sheldrick, 2008; Sheldrick, 2015). Data collection and refinement parameters are shown in Supplementary Table 1. CCDC 1915994-1915996 contain the supplementary crystallographic data for this paper. These data can be obtained free of charge from The Cambridge Crystallographic Data Centre via www.ccdc.cam.ac.uk/structures. Samples of NPSP-795 and AXT-914 crystallised from acetone, and AXT-914 from ethanol, were ground to a fine powder in a mortar and pestle and their powder diffraction patterns recorded using the same equipment.

### Estimation of lung deposited dose, allometric scaling in humans, formulation and drug stability studies

These studies were performed by Cardiff Scintigraphics (Cardiff, United Kingdom) using 1 mM aqueous alcoholic solutions of CaSR NAMs in propylene glycol (PG) followed by a 300-fold dilution into PBS for NPSP-795, Ronacaleret and JTT-305 (final drug concentrations 3 µM; final propylene glycol content 0.3%); AXT-914 was insoluble in propylene glycol, so it was dissolved in propylene glycol 0.27% + ethanol 0.03%. Because of the structural similarities between NPSP-795 and Ronacaleret, further analysis was carried out on only the amino alcohol NPSP-795 and the structurally unrelated quinazolin-2-one AXT-914. For the full methods please see the Supplementary Materials.

Given the volume of the exposure chamber (15.2 l), the inhaled fraction (estimated from the fine particle fraction of which 86.59% of particles were <3μm in diameter), the respiratory minute volume of BALB/c mice (0.024 l/min, determined from a tidal volume of 0.15 ml and a respiratory rate of 160 breaths/min) and the duration of exposure used in the pharmacology studies (60 minutes), this equates to an atmospheric concentration of NPSP-795 of 0.69 µg/l. To correct for the 3 µM concentration used in the pharmacology studies, a value of 0.0069 µg/l is used in the equation. Using these values gives an estimated lung dosage of ~9 ng of NPSP-795 in a BALB/c mouse from nebulisation of a 3 µM solution over 60 min.

The estimated human dosage of NPSP-795 varies from 2 µg using allometric (body surface area) scaling, to 28 µg using theoretical scaled lung weight, 30 µg using scaled body weight and 58 µg using reported lung weights. From these estimates a value of ~30 µg was selected as a basis for the formulation stability studies.

Nebulizer solutions with a concentration of 0.4 mg/ml were prepared since only approximately 10% of the amount of drug in the nebuliser (i.e. 100 µg) is likely to be deposited in the lung in human studies. For solubility and stability testing, 2 mg of NPSP-795 were dissolved in either 100 µl of absolute ethanol (EtOH) or 150 µl of PG and aliquots kept in different storage conditions. Subsequently, one mL of 0.9 % w/v NaCl was added to the NPSP-795 in EtOH or PG and both formulations were subjected to 20 min centrifugation (SciQuip Ltd, SciSpin One Compact Centrifuge) at 2000 rpm before sampling the clear solutions. Approximately 1.5 ml of the EtOH and PG NPSP-795 in NaCl solutions were transferred into 3 separate Eppendorf tubes before storing at ambient room temperature, 5°C and at 40°C / 75 % relative humidity (RH). At each time point 25 µl of this sample were transferred to a 10 ml volumetric flask and diluted to volume with HPLC Recovery Solution (30% acetonitrile). 2 ml samples were transferred to HPLC vials for analysis.

The solubility of 400 µg/ml AXT9-14 in EtOH and PG were comparable and required high concentrations of co-solvent to ensure complete solubility, illustrating the poor solubility of AXT-914 in both, aqueous EtOH or PG solutions. To ensure complete solubility of AXT-914 in aqueous EtOH solutions requires EtOH to be at an approximate minimum concentration of 53% v/v. For toxicology studies, this value would need to be increased to at least 60% v/v to reduce the potential for precipitation during nebulisation. However, as a nebuliser formulation this currently exceeds approved levels for human use. In aqueous PG solutions an approximate minimum concentration of 71% w/w was estimated to ensure complete solubility of AXT-914. For toxicology studies, the use of a 100% PG nebuliser formulation is likely to be needed, indicating that neither EtOH, nor PG solutions can be generated for human studies with AXT-914. Therefore, it may be preferred to formulate AXT-914 as a dry power inhaler formulation.

### AXT-914 micronization and characterization of the milled material

Next, we tested the suitability of AXT-914 for pulmonary administration as a dry powder inhaler, by testing: 1) if AXT-914 can be micronized into particles of respirable size (around 3 µm), 2) if the micronized material is of crystalline, thermally stable, inert and non-hygroscopic. For the full methods please see the Supplementary Materials.

### Quantification of airways remodelling

Quantitative image analysis was performed using StrataQuest image analysis software (TissueGnostics, Austria) (TissueGnostics). An average of 16 smaller airways (<40,000 µm^2^; n = 3-21) were examined from 3-4 Masson’s trichrome stained lung sections from each animal (n=6 per condition) for assessment of remodelling markers within manually identified regions of interest (Supplementary Figure 7A, B). Manual airway object identification was performed by operators blinded to the experimental conditions using the Opensource blindrename.pl script (Slater, 2016).

### Statistical analysis

Statistical analysis was undertaken with GraphPad Prism 7 software (GraphPad Software, USA) and sample size and all analysis steps were decided before the data had been gathered. Data are expressed as mean and SD. Student’s two-tailed, unpaired or paired t tests were used to compare two data sets; one- or two-way ANOVA with an appropriate *post hoc* test as stated in the figure legends was used for multiple comparisons.

## Results

### Determination of CaSR NAM efficacies and potencies

**Figure 1:**
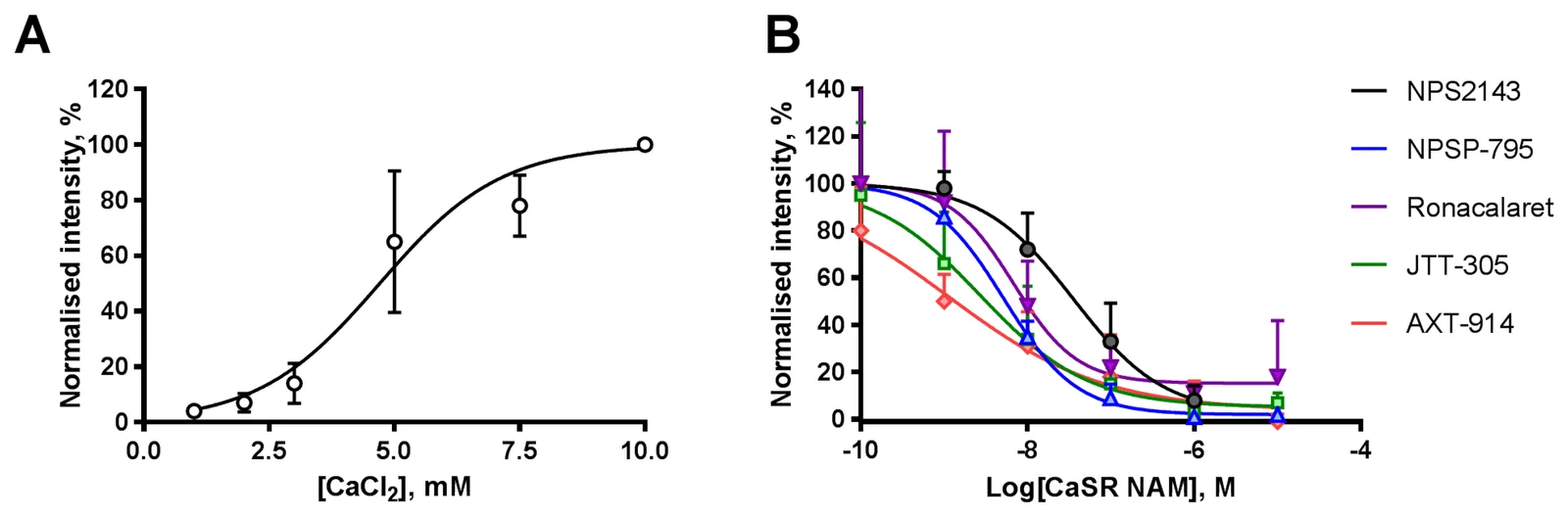
Structurally unrelated amino alcohol (NPSP-795, Ronacaleret, JTT-305 and NPS2143) and quinazolin-2-one (AXT-914) CaSR NAMs inhibit the human CaSR *in vitro*. **A**. Activation of the CaSR in response to increasing Ca^2+^_o_ concentrations (0.5-10 mM) in HEK293 cells stably transfected with the human CaSR. The estimated EC_50_ value is 5 mM. **B**. Inhibitory effects of cumulative additions of CaSR NAMs previously tested in the clinic (NPSP-795, Ronacaleret, JTT-305 and AXT-914) and of the positive control, NPS2143, on CaSR activation evoked by 5 mM Ca^2+^_o_. All CaSR NAMs previously tested in the clinic appear to be more potent than the positive control, with estimated IC_80_ values for NPSP-795, Ronacaleret, JTT-305 and AXT-914 of 20 nM while for NPS2143 IC_80_ is 220 nM. Data are presented as mean ± SD; N = 3-9, n = 125-520, where N is the number of independent experiments, and n is the number of individual cells.

All calcilytics examined concentration-dependently suppressed CaSR-mediated increases in intracellular Ca^2+^ induced by 5mM extracellular Ca^2+^ in HEK-CaSR cells (Figure 1). The CaSR NAMs previously tested in the clinic, NPSP-795, Ronacaleret, AXT-914 and JTT-305, with estimated IC_80_ values of around 20nM, were all ten times more potent than the laboratory-grade NAM, NPS2143 (IC_80_ 202nM; Table 1). Based on lung dosage estimates (see relevant Methods section), and total mouse lung capacity of 1 mL, this may be roughly approximated to stock solutions of 3μM for the clinically tested calcilytics and 25μM for the positive control NPS2143 for *in vivo* experiments.

### CaSR NAMs induce airway relaxation in isolated naïve mouse airways

**Figure 2:**
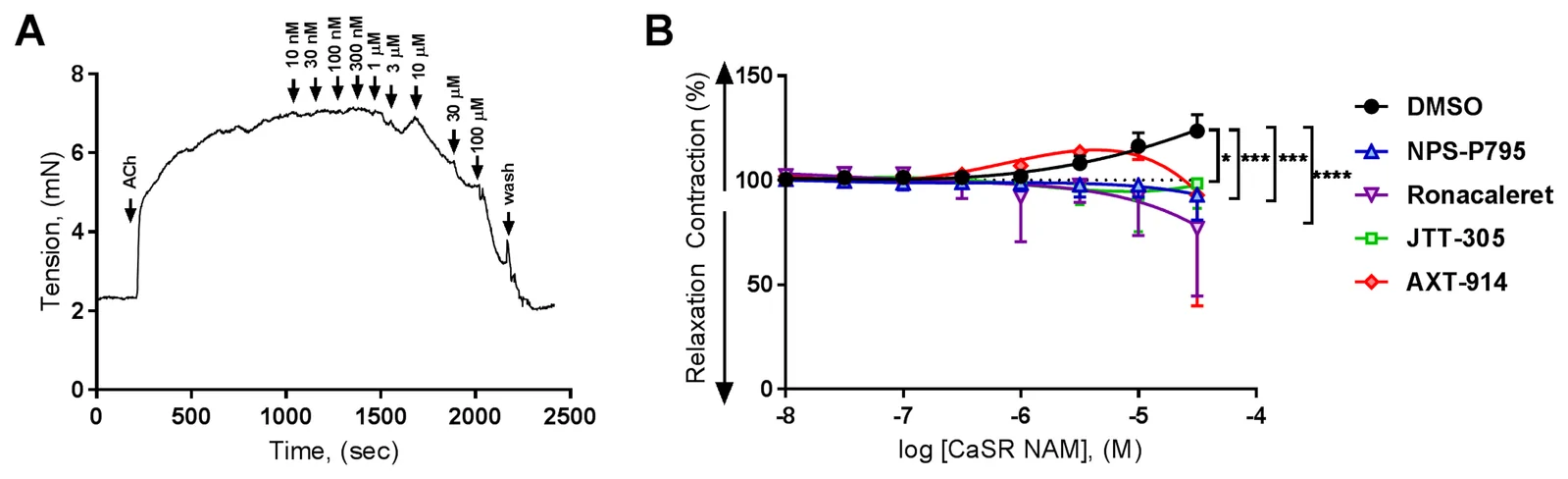
Effects of amino alcohol and quinazolin-2-one CaSR NAMs on tracheal tone. **A**. Exemplar trace representing effects of cumulative application of rising concentrations of Ronacaleret on ACh-induced tone in tracheal rings from naïve BALB/c mice. **B**. Summary data showing effects of DMSO vehicle or CaSR NAMs on the ACh-induced tone in medium containing pro-inflammatory free ionised concentrations (2 mM Ca^2+^). Data are shown as mean ± SD; ANOVA with Dunnett’s *post-hoc* test, * p<0.05; *** p<0.001; **** p<0.0001; N = 3-6 trachea rings from individual animals per each experimental group.

The next steps were to investigate the effects of CaSR NAMs on airways contractility by measuring tension changes of naïve mouse tracheae half-maximally contracted with ACh in medium containing 2 mM Ca^2+^ to mimic pro-inflammatory conditions. Figure 2 shows that the DMSO vehicle applied at the tracheas pre-contracted with ACh at concentrations matching those used to dissolve the CaSR NAMs evoked a concentration-dependent increase in the tracheal tone (up to +23.6±7.8% from the steady-state ACh-induced tone). In contrast, all CaSR NAMs tested relaxed pre-contracted mouse tracheas at concentrations of 100 nM or greater (up to -30.7% (NPSP-795, N=5), -25.2% (JTT-305, N=3), -46.8% (Ronacaleret, N=5),-30.8% (AXT-914, N=4) at 30 μM) from the corresponding level of tone induced by the DMSO vehicle). CaSR NAMs were still able to relax precontracted tracheas in the presence of medium containing physiological Ca^2+^ concentrations (*i.e*., 1 mM) albeit less effectively than in pro-inflammatory conditions (Supplementary Figure 1). No CaSR NAM evoked constriction in the presence of medium containing either physiological (Supplementary Figure 2) or pathological (Figure 2) Ca^2+^ levels.

### Topical delivery of structurally unrelated inhaled CaSR NAMs suppresses AHR and inflammation in mice

**Figure 3:**
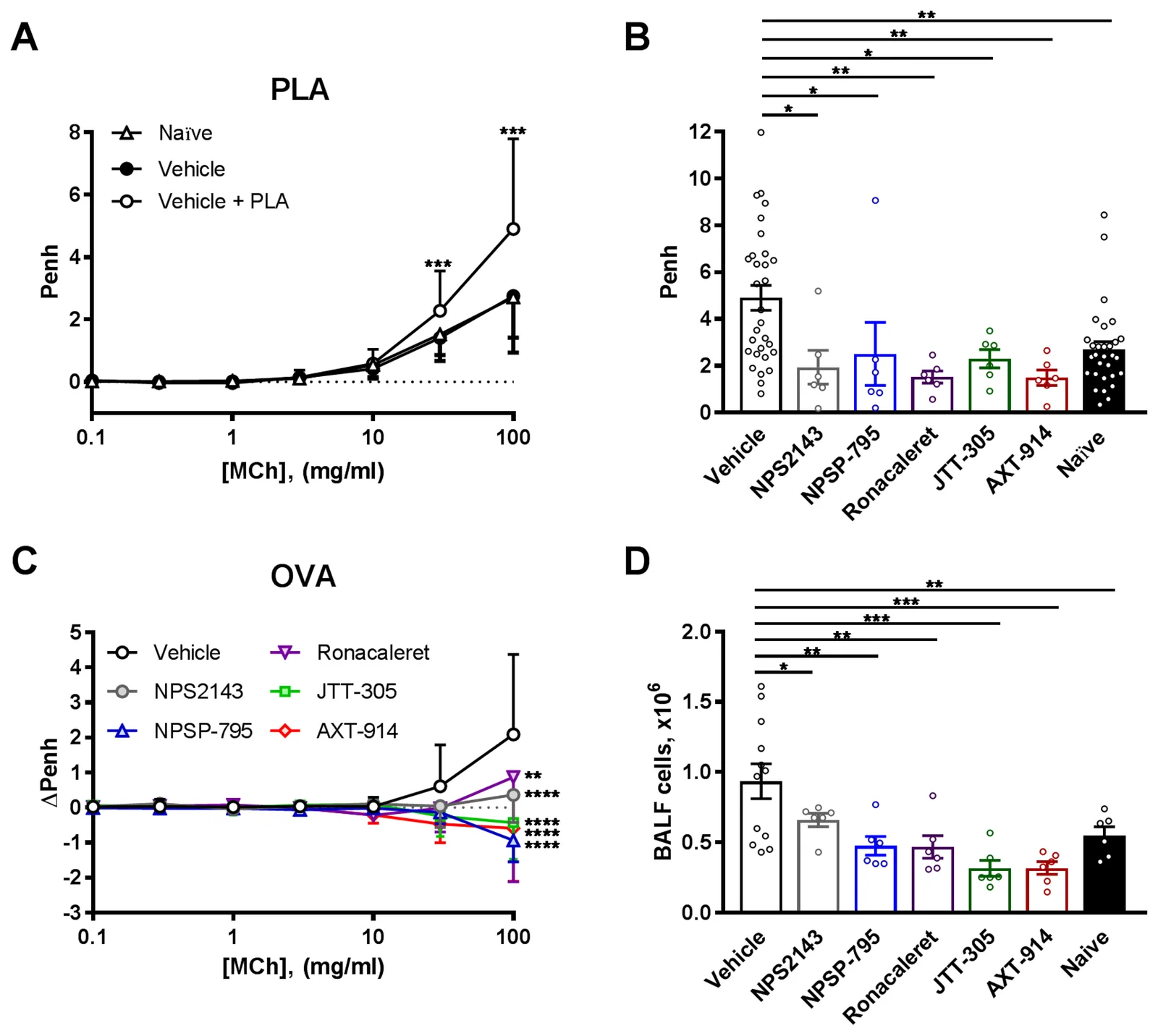
All CaSR NAMs previously tested in the clinic, delivered topically, suppress AHR and inflammation to a comparable extent as the positive control, NPS2143, *in vivo*. **A**. The nebulised CaSR agonist, PLA, evokes a significant increase in airways obstruction (measured as Penh) in MCh-challenged mice. **B**. Nebulised I_max_ doses of CaSR NAMs previously tested in the clinic (NPSP-795, Ronacaleret, JTT-305 and AXT-914) and of the positive control (NPS2143), estimated from *in vitro* experiments, prevent PLA-induced AHR to 100 mg/ml MCh. **C**. Changes in airways obstruction before and after CaSR NAM treatment (Delta (Δ) Penh) in a murine surrogate of allergic asthma. **D**. Effect of CaSR NAM inhalation on inflammatory cell infiltration into the BALF of mice (same animals as in **C**). Vehicle control: 0.1% DMSO in PBS. Data are presented as mean ± SD (A,C) or as scatter dot plot ± SD (B,D); N = 6 animals per experimental group. Statistical comparisons: ANOVA with Dunnett’s (**A**,**C**) or Holm-Sidak’s (**B**,**D**) *post-hoc* tests, * p<0.05; ** *p*<0.01; *** *p*<0.001;**** *p*<0.0001 *vs*. vehicle.

As previously demonstrated (Yarova et al., 2015), exposure of naive mice to the polycation PLA increased MCh responsiveness (Figure 3A) compared with vehicle-treated animals. All nebulized CaSR NAMs (3 μM in the nebulization solution) abolished this effect of PLA (Figure 3B). This effect was concentration-dependent (Supplementary Figure 2). In addition, in line with our previous studies using laboratory-grade compounds (Yarova et al., 2015), prophylactic inhalation of all the tested CaSR NAMs also suppressed AHR and BALF inflammatory cellular infiltration in OVA-sensitised and challenged mice when administered prior to and after OVA challenge in the murine surrogate (Figure 3C,D).

### Effects of CaSR NAMs on the L-type Ca channel

Previous studies have confirmed that CaSR NAMs are deemed safe in the clinic when delivered systemically. Since some CaSR NAMs are structural derivatives of dihydropyridines (L-type Ca^2+^ channel blockers), we explored whether the CaSR NAMs employed in our studies untowardly affect inward, whole-cell macroscopic currents conducted through voltage-gated L_Ca_ channels, which could account for the observed CaSR NAM mediated airway relaxation. Supplementary Figure 3 shows that the CaSR NAMs NPSP-795, Ronacaleret and AXT-914 (all 10 μM), had no effect on normalised L_Ca_ current compared to that measured in the presence of DMSO vehicle whereas, as expected, nifedipine (10 µM), a classical inhibitor of L_Ca_ channels, completely abolished normalised L_Ca_ current (p<0.0001; N = 17).

### Determination of the systemic effects of repeated exposure to inhaled CaSR NAMs

**Figure 4:**
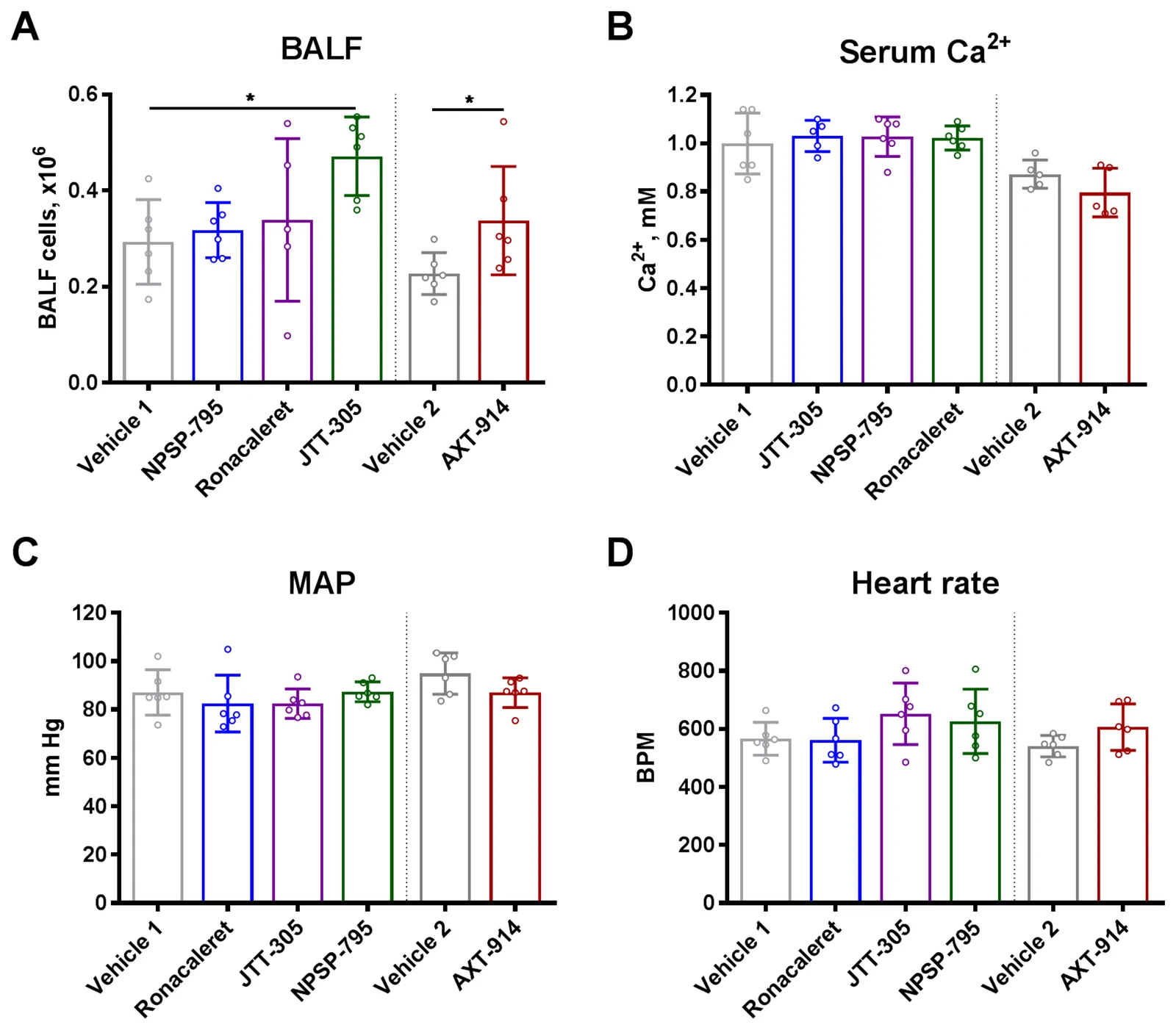
Systemic effects of repeated exposure to CaSR NAM inhalations in naïve mice *in vivo*. Effects of repeated (5 days) exposure to inhaled CaSR NAMs previously tested in the clinic as oral drugs on (**A**) BALF cellular infiltration; (**B**) serum Ca^2+^ concentration; (**C**) mean arterial blood pressure (MAP); (**D**) heart rate (beats per minute: BPM). Vehicle 1: 0.3% propylene glycol in PBS. Vehicle 2: 0.27% propylene glycol + 0.03% ethanol in PBS. Data are shown as scatter dot plot ± SD; N = 5-6 animals per experimental group. Statistical comparisons: ANOVA with Holm-Sidak’s *post-hoc* test (amino alcohol CaSR NAMs, NPSP-795, Ronacaleret and JTT-305) or two-tailed unpaired T-test (quinazolin-2-one CaSR NAM, AXT-914), * *p*<0.05, ** *p*<0.01.

Next, we investigated the effects of repeated exposure of naïve BALB/c mice (5 days, 1 hr/day) to nebulised solutions of the inhaled CaSR NAMs previously tested in clinical trials, and their effects on blood pressure, heart rate, serum Ca^2+^ concentration (a surrogate for changes in plasma PTH) and airways irritation, as reflected by BALF inflammatory cellular infiltration. Repeated administration (5 days, 1 hr/day) of AXT-914 and JTT-305, but not NPSP-795 or Ronacaleret, evoked an increase in the mean BALF total cell count when compared with their respective vehicle controls (from 22.7x10^6^±4.4x10^6^ to 33x10^6^±11.3x10^6^ cells for AXT-914, and from 29.3x10^6^±8.8x10^6^ to 47.15x10^6^±8.1x10^6^ cells for JTT-305; N=6; Figure 4A). No compound caused changes in mean arterial blood pressure, heart rate or serum Ca^2+^ concentration (Figure 4B-D), suggestive of minimal systemic drug overspill into the systemic circulation.

### In vivo PK of CaSR NAMs

**Figure 5:**
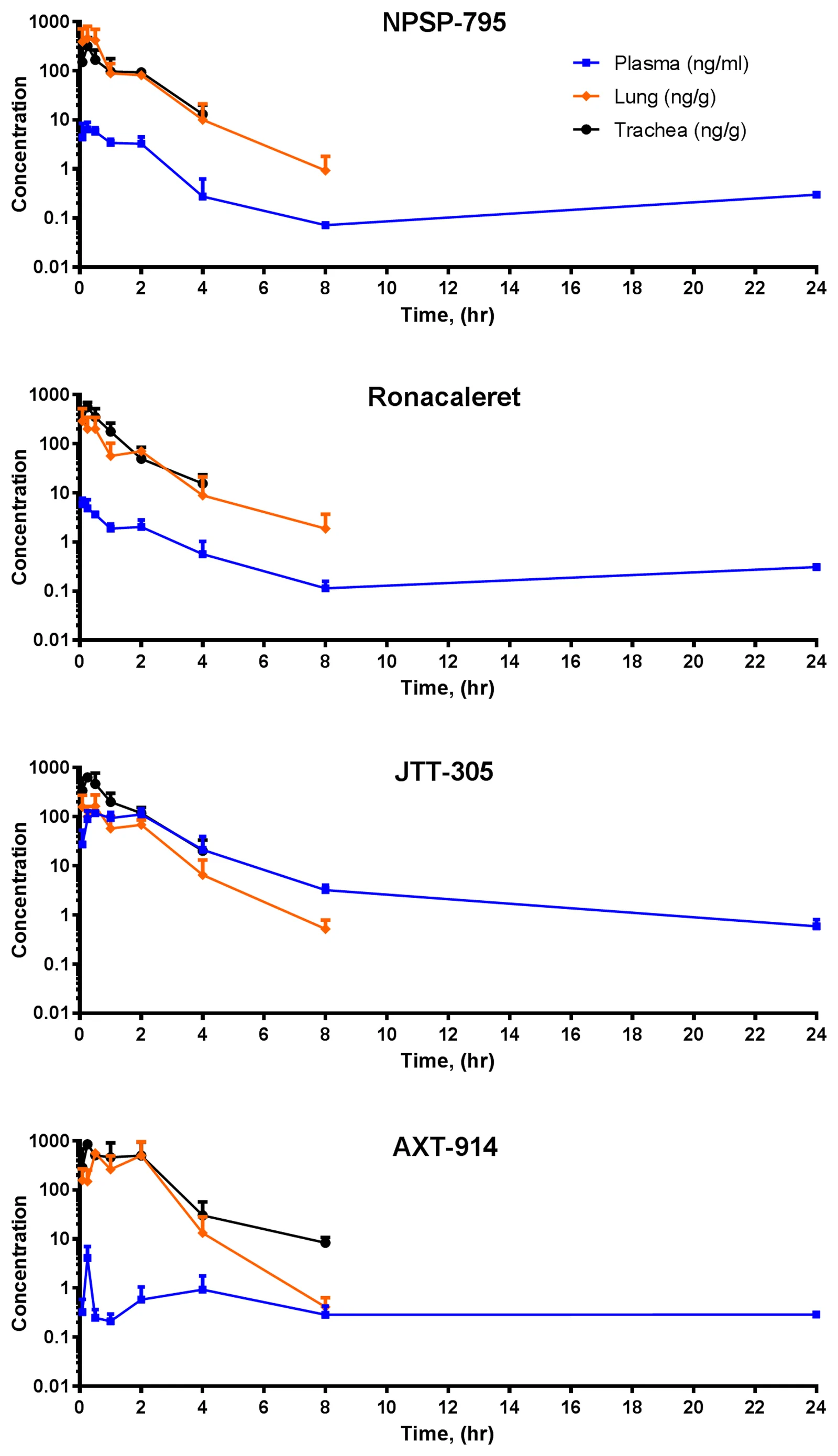
Pharmacokinetics of CaSR NAMs in naïve mice *in vivo*. Retention times in murine blood plasma (blue), lung (orange), and trachea (black) following intra-tracheal instillations of CaSR NAMs previously tested in the clinic. Compounds were re-suspended in 3% DMSO, 97% (5% EtOH/5% glucose in water) at a concentrations of 17.5µg/kg, then filtered through a 0.45 μm pore filter and the filtered solution was administered to the mice. Data are shown as the mean ± SD; N = 1-3 animals per each time point.

Direct intra-tracheal instillation of the four clinically tested CaSR NAMs resulted in an early peak in lung concentrations followed by an exponential decay and clearance from the lung tissue and circulation within 8 hr (Figure 5). NPSP-795, Ronacaleret and AXT-914 exhibited very similar PK profiles although AXT-914 was retained in the trachea for up to eight hours. JTT-305 showed considerably prolonged retention in the circulation, resulting in a low lung/plasma ratio (Table 1). These observations detract from the potential suitability of JTT-305 for topical therapy, and this compound was therefore excluded from further studies.

### PD of inhaled NPSP-795 and AXT-914

**Figure 6:**
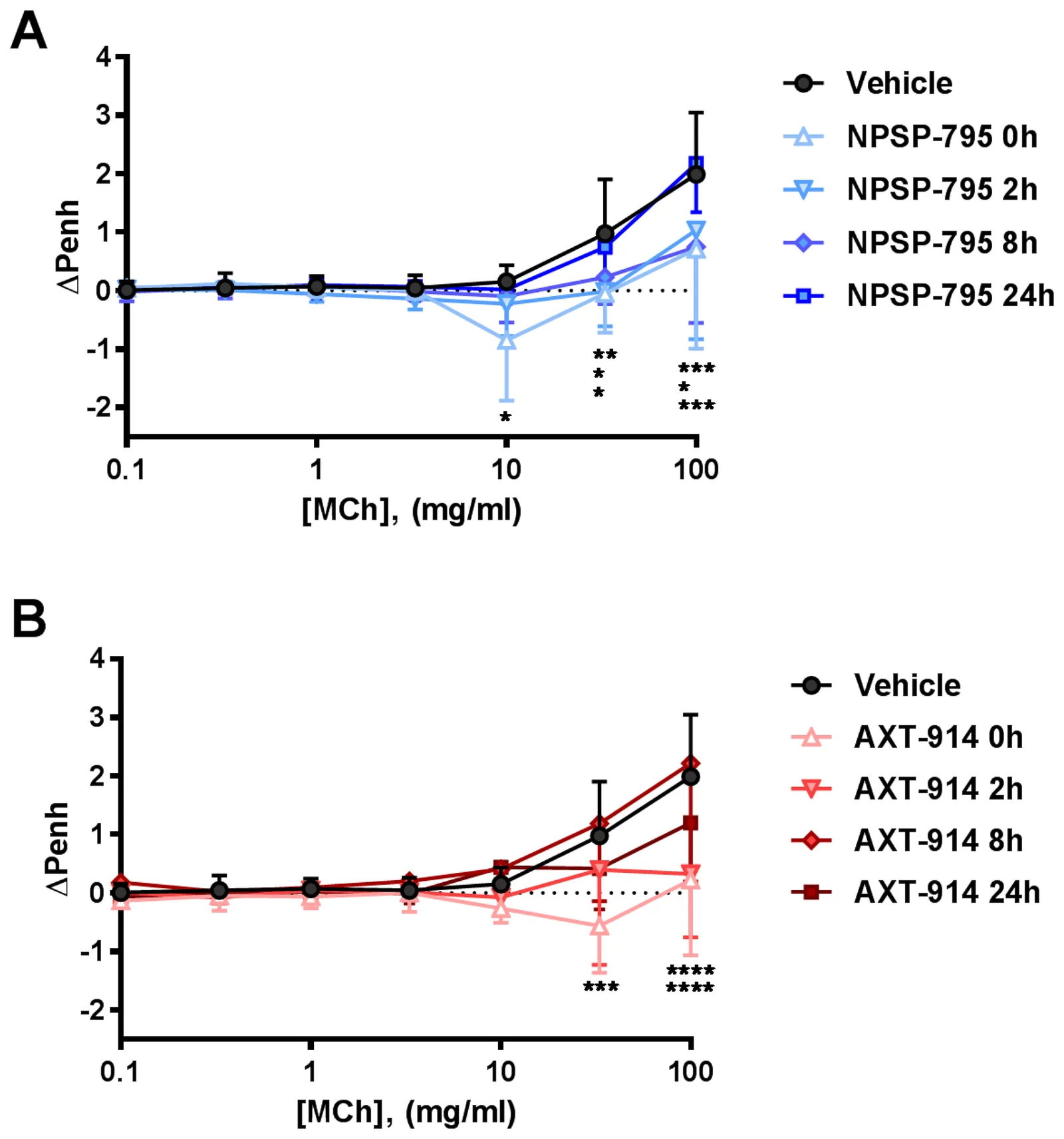
Pharmacodynamics of amino alcohol (NPSP-795) and quinazolin-2-one (AXT-914) CaSR NAMs in naïve mice *in vivo*. Effects of NPSP-795 (**A**) or AXT-914 (**B**) pre-treatment (0, 2, 8 or 24hours) on PLA-induced increase in ΔPenh. Vehicle: 0.1% DMSO in PBS. Data are shown as mean ± SD; N = 5-9 animals per experimental group. Statistical comparisons: ANOVA with Dunnett’s *post hoc* test, * *p*<0.05; ** *p*<0.01; *** *p*<0.001, **** *p*<0.0001.

Current inhaled treatment regimens for asthma are usually based on twice daily dosing, and it would be beneficial if those for inhaled CaSR NAMs were equivalent or better. We investigated the PD of inhaled CaSR NAMs *in vivo* using the acute PLA model described above. As JTT-305 had low lung/plasma ratio, thus is unlikely to be suitable for asthma treatment, and the amino alcohols NPSP-795 and Ronacaleret had very similar chemical structure, PK, potency and efficacy profiles, this study was restricted to the structurally unrelated NPSP-795 and AXT-914. Nebulized CaSR NAM (3 μM) or vehicle was delivered at times zero, one, two, four and 24 hours prior to PLA inhalation (6 μM for 30 min), which was immediately followed by MCh challenge. NPSP-795 abrogated diminished PLA-induced AHR for up to eight hours after treatment (Figure 6A), but was ineffective by 24 hours. AXT-914 abrogated PLA-induced AHR two, but not eight hours or more following nebulised delivery (Figure 6B).

### Crystallography of CaSR NAMs

The amino alcohol NPSP-795 crystallised from acetone as the hydrochloride (Supplementary Figure 4, Supplementary Table 1). Powder diffraction showed that the crystal structure was consistent with the bulk of the recrystallised sample. A later crop of crystallisation material revealed the presence of an additional but unidentified phase which could be a new polymorph or a solvate. Grinding a sample of the pure material resulted in some loss in crystallinity, but no phase transformation (Supplementary Figure 4).

The amino alcohol Ronacaleret crystallised from acetone as the hydrochloride and the crystal structure of obtained was the same as that previously reported (Supplementary Table 1)(Vogt et al., 2014).

The crystals of AXT-914 obtained from acetone revealed the existence of a new single phase before and after grinding (Supplementary Figure 5A/5B, Supplementary Table 1), consisting of four independent molecules with a range of conformations. Comparison of the powder x-ray diffraction data recorded for the sample with the pattern simulated from the single crystal data showed that the sample was a single phase. The powder pattern recorded for the sample before recrystallisation indicated the existence of a different but yet to be identified phase, possibly a polymorph or solvate. Grinding the recrystallised sample did not reveal a change in phase (Supplementary Figure 5B). Crystals of AXT-914 existed in two distinct phases according to whether they had been crystallised from ethanol or acetone. Both were resistant to grinding (Supplementary Figure 5C).

### Formulation studies for the amino alcohol NPSP-795 and the quinazolin-2-one AXT-914 CaSR NAMs

For NPSP-795, the data provide confidence that a simple nebulizer solution can be prepared for use by a clinical research organisation, and the stability of such solutions will permit daily dosing in a standard schedule. Follow-on studies have shown physical stability in 5% solutions of either EtOH or PG with chemical stability for at least 6 hours when refrigerated, which is compatible with human use.

In contrast, AXT-914 demonstrates poor solubility of in both aqueous EtOH or PG solutions and, for routine use in humans would likely require formulation either as a nebuliser suspension or as a dry power inhaler formulation. In both circumstances, the AXT-914 powder would need to be micronized to around 2-3 µm before formulation studies could proceed. Therefore, the next steps were to investigate the possibility that AXT-914 could be micronized into particles of respirable size.

### AXT914 micronization, particle size determination and stability

The particle size was assessed to ensure adequate size for lung deposition, with an average particle size from 1 to 6 μm. After jet-milling AXT-914, using laser diffraction, a monodispersed distribution could be observed (Supplementary Figure 6A, left), with D(50) of 3.59 μm (Supplementary Figure 6A, right). The size of both raw and micronized particles was confirmed using SEM, as shown in Supplementary Figure 6B. Using the SEM scale tool, 27 random particles were measured and ranged from 1.04 to 4.38 μm, with average size of 2.22±0.75 μm. The thermal response of the milled material showed an endothermic peak at 125°C indicative of the melting temperature of AXT-914, while the exothermic peak at 270°C may be related to the thermal degradation of the substance (Supplementary Figure 6C). Representative moisture sorption isotherms as a function of relative humidity (%) are shown in Supplementary Figure 6D. A minimal weight gain of 0.2% was observed between 0 and 90% RH, indicating that milled AXT-914 is crystalline.

### Head-to-head comparison of the anti-inflammatory activities of inhaled CaSR NAM and inhaled corticosteroid in a chronic murine asthma surrogate

**Figure 7:**
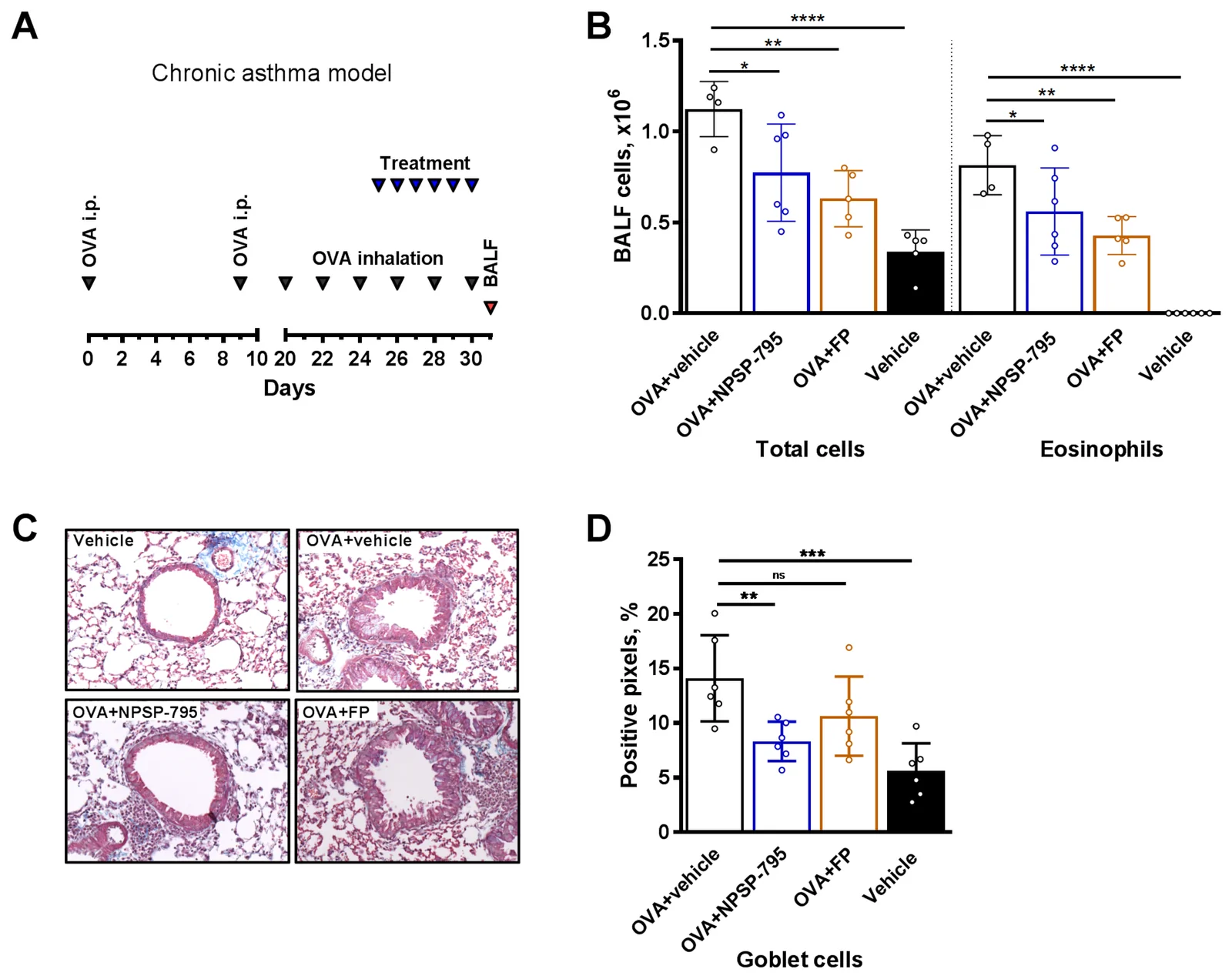
Head-to-head comparison of inhaled CaSR NAMs or FP treatment on BALF inflammation and remodelling in a therapeutic asthma model. **A**. Schematic exposure protocol. **B**. Balbc mice were sensitised and challenged with OVA and the effects of inhaled NPSP-795, or inhaled FP, determined on BALF total leucocyte (left panel) and eosinophil cell count (right panel) at the end of the experiment. N = 4-6 animals per experimental group. **C**. Representative images of Masson’s trichrome stained lung sections. **D**. The effects of inhaled NPSP-795 or FP on goblet cells were quantified using StrataQuest image analysis software as described in Supplementary Figure 7. Data are shown as scatter dot plot ± SD, with N = 6 animals per experimental group, and n = 3-21 airways from 3-4 Masson’s trichrome-stained lung sections per animal. Statistical comparisons: 1-way ANOVA with Holm-Sidak’s *post hoc* test, * *p*<0.05; ** *p*<0.01, *** *p*<0.001, **** *p*<0.0001.

Because the CaSR NAM NPSP-795 is suitable for nebulization while AXT-914 requires the development of a dry powder inhaler before further testing in rodent models and in humans can be undertaken, NPSP-795 alone was used in the head-to-head comparison against the current standard of care experiments. We developed a murine asthma surrogate based on OVA sensitisation and challenge (Figure 7A) in which we tested the effects of repeated exposure to inhaled NPSP-795 against those of the standard-of-care, fluticasone propionate (FP). Concomitant, repeated exposure to nebulised NPSP-795 or FP during the later phases of serial OVA inhalation challenge in the OVA sensitised mice, which was associated with a significant elevation of the mean numbers of total cells and eosinophils in the BALF, significantly reduced the mean total cell and eosinophil counts to a comparable degree (Figure 7B). Quantitative image analysis revealed that NPSP-795 inhalation reduced the mean goblet cell number in the airways whilst FP did not (Figure 7C). Other remodelling parameters such as total tissue, epithelial tissue, and airway size were all unaffected (Supplementary Figure 7C,D).

## Discussion

In our previous study (Yarova et al., 2015) we presented data compatible with the hypothesis that bronchial smooth muscle hyperresponsiveness, which is the quintessential feature of human asthma, is caused, at least in part, by elevated expression and/or activation of the CaSR on airways smooth muscle cells, resulting in a heightened response to a contractile stimulus, and that blockade of the CaSR with CaSR NAMs, also known as calcilytics, abolish it. Here we address the practicability of repurposing a range of NAMs previously tested in clinical trials of osteoporosis therapy and administered systemically for their suitability for topical application for asthma therapy using dosing regimens and inhaler-delivered, topical formulations resembling those in current clinical use. To this end we employed animal surrogates in which the CaSR on airways smooth muscle cells was stimulated artificially with nebulised PLA, a CaSR activator, or where animals were sensitised and challenged with OVA, to create asthma-like airways inflammation.

In many asthmatics, bronchial smooth muscle hyperresponsiveness is further complicated by the co-existence of airways inflammation. Our earlier studies also revealed that products of inflammatory cells typically involved in airways inflammation in asthma, such as eosinophils and neutrophils, may directly exacerbate bronchospasm in patients with bronchial smooth muscle hyperresponsiveness by releasing polycations (e.g. eosinophil cationic proteins, major basic proteins or polyamines), which are direct agonists at the CaSR, (Yarova et al., 2015). This is a previously known, but mechanistically unexplained positive feedback mechanism for asthma exacerbation by the products of inflammatory cells, which we have shown is abolished by topical CaSR NAM therapy (Yarova et al., 2015; Corrigan, 2020). In addition, inflammation, by causing swelling of the airways lining with oedema, greatly amplifies the obstruction caused by a given degree of smooth muscle contraction. Topical and systemic corticosteroids ameliorate asthma not by targeting bronchial smooth muscle hyperresponsiveness, but by reducing infiltration of the airways with inflammatory cells which produce CaSR agonists and by reducing oedema and swelling of the airways mucosa, thus increasing their internal diameter (Chan and Silverman, 1993; Baraldi et al., 2005; Carraro et al., 2010). Corticosteroids may also enhance the effects of bronchodilators (Koziol-White et al., 2020). Studies suggesting that corticosteroids reduce hyperresponsiveness of the asthmatic airways over time (Lundgren et al., 1988; Jeffery et al., 1992; Laitinen and Laitinen, 1995) most likely reflect the fact that they reduce airways mucosal inflammation and oedema progressively and prophylactically in those asthmatics who evince a significant amount of this inflammation, although are ineffective against the underlying phenomenon of bronchial smooth muscle hyperresponsiveness. These data suggest that inhaled CaSR NAMs represent the first anti-asthma therapy with the potential not only to abolish bronchial smooth muscle hyperresponsiveness (and not simply temporarily antagonise its effects, as seen with bronchodilators (Bourke et al., 2019)), but also to reverse bronchial mucosal inflammation and oedema and the local over-production of CaSR agonists by inflammatory cells. Thus, they offer the possibility of much more comprehensive asthma control with a single, non-toxic, non-steroidal topical drug.

The CaSR is widely expressed on structural and inflammatory cells of the airways. CaSR NAMs function as upstream inhibitors of many signalling processes including those that result in the release of inflammatory mediators (Lee et al., 2012; Yarova et al., 2015; Lee et al., 2017a; Lee et al., 2017b). In the present study we show that a range of CaSR NAMs previously tested in clinical settings, administered directly to the airways, abolish PLA-induced AHR and inhibit the airways inflammatory response associated with OVA sensitisation and challenge at least as well as topical corticosteroids. This highlights a class-specific effect of these drugs and firmly supports the hypothesis that the observed anti-inflammatory properties reflect blockade of the CaSR. In addition, NPSP-795 reduces goblet cell hyperplasia in a chronic asthma model. These results concur with previously published observations that CaSR NAMs suppress mucus secretion in tobacco smoke-stimulated human epithelial cells (Lee et al., 2017b), suggesting that chronic use of inhaled CaSR NAMs might preserve airway structure over time and prevent irreversible airways blockage through remodelling. Noteworthy, CaSR NAMs evoke small relaxation of isolated murine airways independently of their class, and this relaxation is not due to the off-target inhibition of L-type Ca^2+^ channels.

Systemic CaSR NAMs were developed to evoke pulsatile changes in plasma PTH, an established bone anabolic stimulus. Despite good safety and tolerability profiles in osteoporosis clinical trials (Widler, 2011; Halse et al., 2014), some patients developed hypercalcaemia. To obviate this issue, topical, inhaled CaSR NAM therapy for asthma should ideally result in topically effective airways concentrations with minimal systemic absorption (Lotvall, 1997). Our data, albeit in laboratory animals, suggest that topical application of the clinical grade CaSR NAMs in concentrations likely to be therapeutically relevant for asthma therapy does not derange calcium metabolism or exert any detectable cardiorespiratory unwanted effects. Repeat exposure of naïve mice to either NPSP-795 or Ronacaleret did not cause airway irritancy, while JTT-305 and AXT-914 slightly increased the mean numbers of cells in the BALF of naïve animals, suggesting a possible effect on cellular migration or capillary permeability following drug nebulisation of these two compounds when applied topically to the airways. However, this potential issue might putatively be overcome by delivery using conventional, anti-asthma metered-dose inhalers or dry powder, microparticle suspensions as distinct from nebulised solutions.

In PK studies in mice, NPSP-795, Ronacaleret and AXT-914 were cleared from the lungs within 8 hours with minimal systemic exposure after intra-tracheal dosing, indicating that in humans these molecules might be suitable for twice daily, topical application. In contrast, JTT-305 showed substantial retention in the circulation, detracting from its suitability for topical repurposing. In addition, our data confirm lack of off-target effects of NPSP-795, Ronacaleret and AXT-914 at the dihydropyridine-sensitive L-type Ca^2+^ channels expressed in the cardiovascular system (Bodi et al., 2005).

Since NPSP-795 and Ronacaleret are structurally very similar, further studies were carried out using the amino alcohol NPSP-795 and the quinazolin-2-one AXT-914. Pharmacodynamically, NPSP-795 abrogated PLA-induced bronchial smooth muscle hyperresponsiveness for at least 8h following topical application, but was ineffective (presumably reflecting elimination) within 24h. AXT-914 in contrast exhibited a shorter duration of action, although PK studies demonstrated that it was retained longer in the trachea. Formulation studies provide confidence that for NPSP-795 a simple nebuliser solution can be prepared for first-in-human studies in conditions typically found in a clinical research organisation, and the stability of such solutions will permit daily dosing in a standard schedule. The estimated human dosage for NPSP-795 is 30 µg, which is comparable to many other inhaled asthma drugs.

In contrast, AXT-914 is insoluble in solvents commonly used in clinical settings, requiring further formulation development. Crystallization and micronization studies show that AXT-914 retains a single phase in different crystallisation solvents, and it can be milled into particles of respirable size ranging between 2-3 μm, thus it is potentially suitable for inhaled delivery. The structure of the jet-milled AXT-914 is crystalline and chemically stable, indicating suitability of the micronized material as a nebulized suspension, or a dry powder formulation.

In summary, these pre-clinical data support the hypothesis that NPSP-795, and AXT-914, after formulation refinement, have suitable pharmacological, PK/PD and safety profiles appropriate for topical administration to human patients with asthma. Current management of refractory asthma requires therapy with bronchodilators and topical and systemic anti-inflammatory corticosteroids, and recently “biological” therapies, all of which raise concerns or questions of safety, efficacy, unwanted effects, compliance, logistics and cost. Successful delivery of CaSR NAMs to the airways promises to eliminate bronchial smooth muscle hyperresponsiveness and thus spontaneous constriction of the asthmatics airways, rendering routine use of bronchodilators redundant, and inhibiting airways inflammation in a corticosteroid-independent fashion. In addition, long term use of topical CaSR NAM therapy may prove to alter the natural history of irreversible airways obstruction.

In conclusion, inhaled CaSR NAMs could provide a first-in-class, single topical therapy for asthma in children and adults, simultaneously eliminating airways smooth muscle hyperresponsiveness and reducing airways inflammation and remodelling safely, efficiently and in cost-effective manner.

## Supplementary Material

Supplementary Material

## Figures and Tables

**Table 1:** Compound characteristics, *in vitro* potencies and *in vivo* PK parameters for all tested calcilytics. IC_50_ and IC_80_ values were calculated by nonlinear regression (variable slope) of concentration-response curves to CaSR NAMs obtained using measurements of intracellular Ca^2+^ as a readout for CaSR activation (by Ca^2+^_o_) and inhibition (by CaSR NAMs) in HEK-CaSR. Plasma and lung maximal concentrations (C_max_), time of maximal concentration (T_max_), time of 50% maximal concentration (t_1/2_), area under the curve (AUC_all_), and lung:plasma ratio were obtained in mice after intra-tracheal instillations of the tested compounds (nominal concentrations of each compound: 17.5 µg/kg).

	NPSP-795	Ronacaleret	JTT-305	AXT-914
MW	436.5	447.5	514	487.4
FW	473	483.9	563	487.4
Salt	HCl	HCl	0.5 H_2_SO_4_	-
IC_50_, nM	5.2	6.8	2.7	1.1
IC_80_, nM	21.1	23.0	21.2	22.1
Plasma t_1_/_2_, h	1.1	1.5	3.4	2.4
Plasma T_max_, h	0.3	0.3	0.5	0.3
Plasma C_max_, ng/mL	6.4	4.9	118.0	4.2
Plasma AUC_all_, ng·h/ml	12.4	9.3	404.1	5.3
Lung t_1/2_, h	1.0	1.2	0.9	0.6
Lung T_max_, h	0.3	0.1	0.5	0.5
Lung C_max_, ng/g	457.5	287.3	164.0	560.3
Lung AUC_all_, ng·h/g	511.1	331.6	269.3	1250.5
Lung:Plasma Ratio (AUC_all_)	41.2	35.7	0.7	234.0

**Table 2:** CaSR NAMs previously developed for systemic use and tested in humans potentially available for repurposing as inhaled asthma drugs. Development compound table illustrating compound code, development company, chemical structure, molecular weight (MW), crystal structures, developmental stages and number of patients, duration and references to the clinical trials. NPSP-795, Ronacaleret, JTT-305 and AXT-914 crystal structures were generated as part of the current studies.

Code	Company	MW (g/mol)	Crystal structure; PubChem CID	Development stage	StdInChlKey; NCT; References
**NPSP795/SB-423562** (amino-alcohol) NPSP790/ SB-423557 (ester pro-drug) NPSP795/SHP635 (amino-alcohol)	NPS/GSK (osteoporosis) NPS (ADH1)	436.5	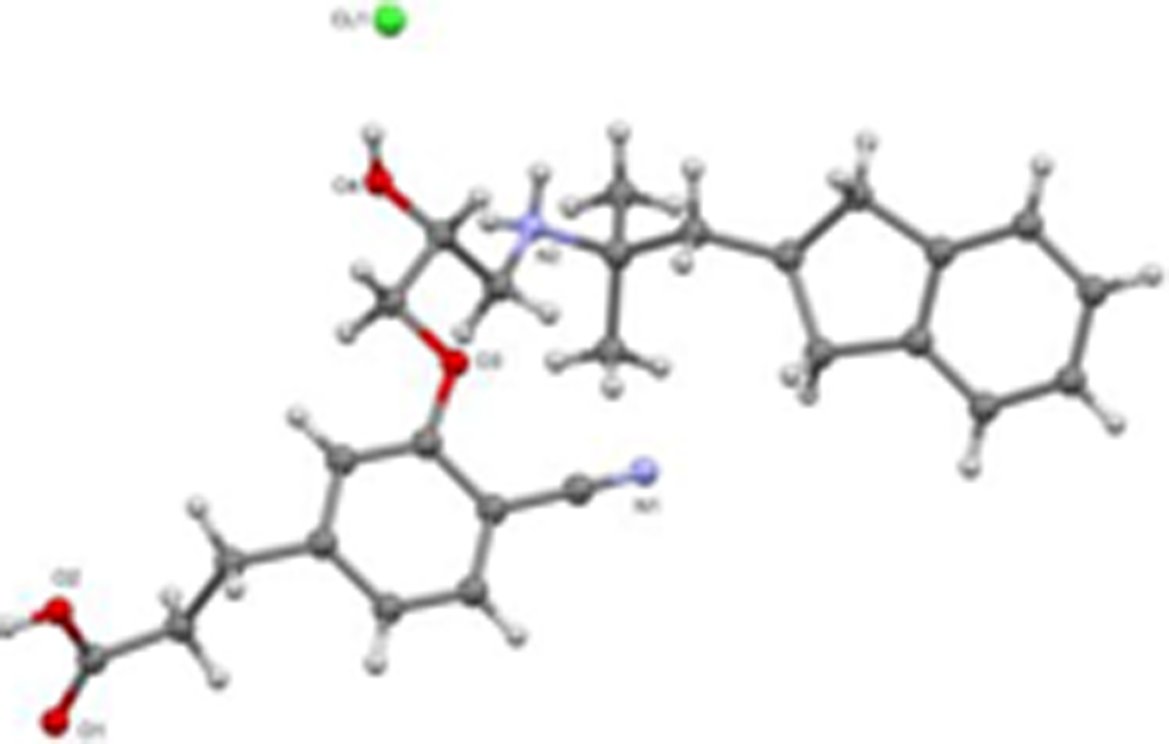 **9910902**	Phase I (n=28; i.v.; 2 study sessions seven days apart) (n=50; oral; > 2 study sessions seven days apart) Phase II (n=7; 17 days; i.v.)	NJBFJCJKWWIKRD- HSZRJFAPSA-N; https://pubmed.ncbi.nlm.nih.gov/19786130 NCT02204579; https://pubmed.ncbi.nlm.nih.gov/31063613
**Ronacaleret/SB-** **751689** (amino-alcohol)	GSK (osteoporosis)	447.5	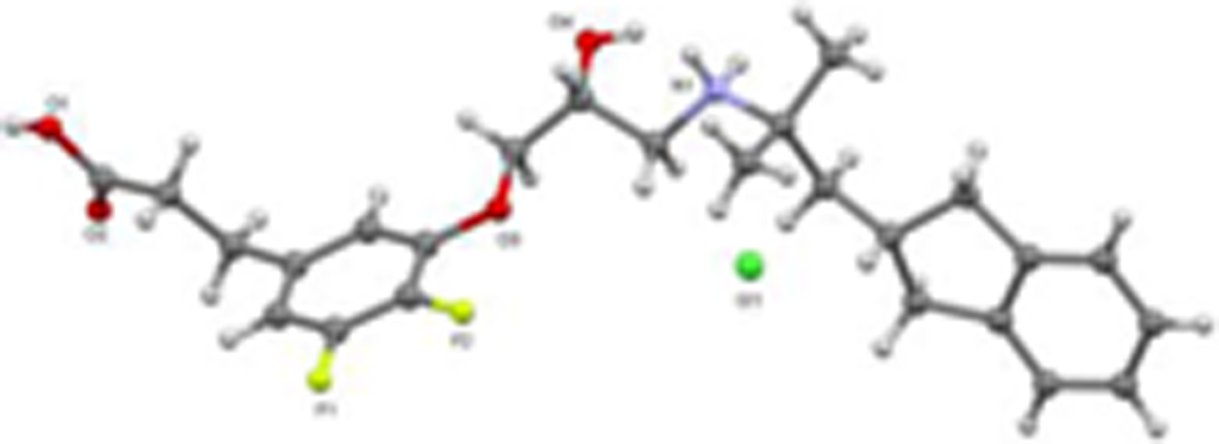 **10345214**	Phase II (n=569; 12 months; oral)	FQJISUPNMFRIFZ- HXUWFJFHSA-N; NCT00471237; https://pubmed.ncbi.nlm.nih.gov/23756230
**JTT-305/MK-5442;** Encaleret (amino alcohol)	Japan Tobacco/Merck (osteoporosis)	514	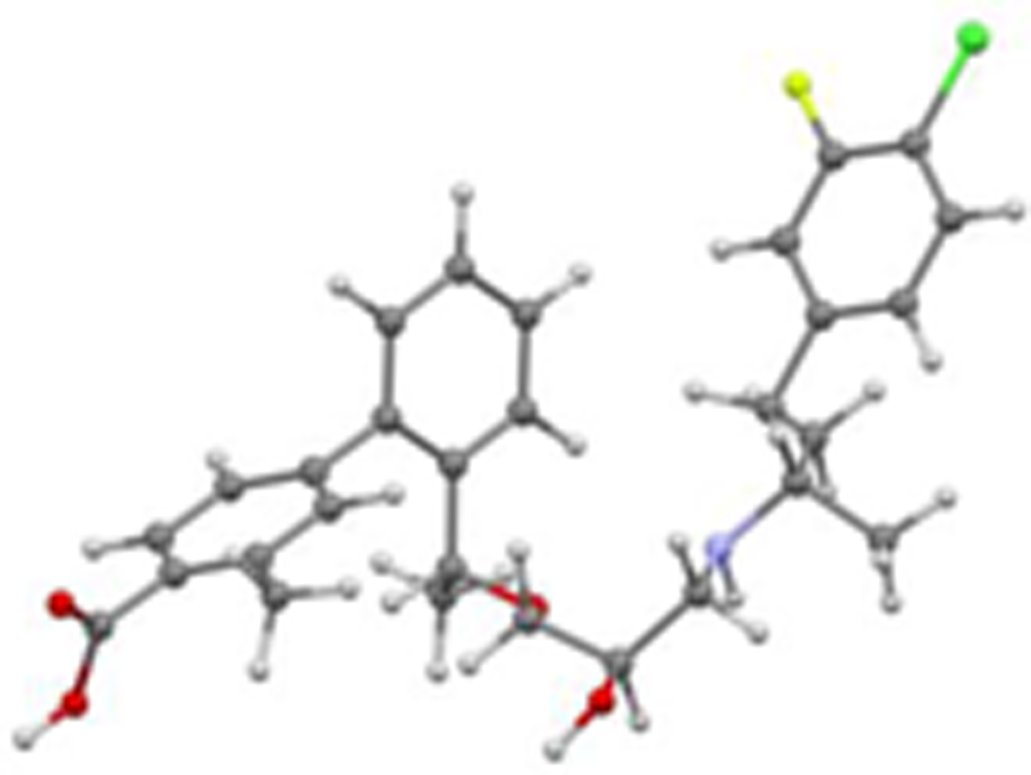 **46917559**	Phase II (n=526; 12 months; oral)	UNFHDRVFEQPUEL- DENIHFKCSA-N; NCT00996801; https://pubmed.ncbi.nlm.nih.gov/26556736
**AXT914** (quinazolin-2-one)	Novartis (osteoporosis)	487.4	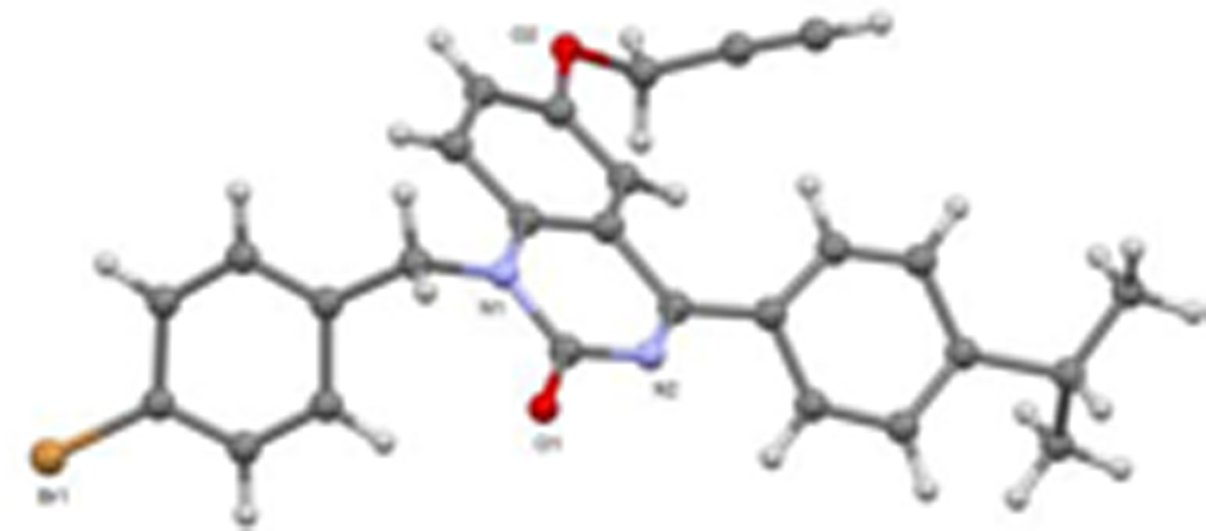 **n.a.**	Phase II (n=105; 4 weeks; oral)	NCT00417261; https://pubmed.ncbi.nlm.nih.gov/24769332

## Recommended section

Drug Discovery and Translational Medicine

## Significance statement

CaSR NAMs reduce airways smooth muscle hyperresponsiveness, reverse airways inflammation as efficiently as topical corticosteroids, and suppress airways remodelling in asthma surrogates. CaSR NAMs, initially developed for oral therapy of osteoporosis proved inefficacious for this indication despite being safe and well tolerated. Here we show that structurally unrelated CaSR NAMs are suitable for inhaled delivery and represent a one stop, steroid-free approach to asthma control and prophylaxis.

## Non-standard abbreviations

ACh: acetylcholine
AHR: airway hyperresponsiveness
BALF: bronchoalveolar lavage fluid
CaSR: extracellular Ca^2+^/cation-sensing receptor
DSC: Differential scanning calorimetry
DVS: dynamic vapour sorption
FP: fluticasone propionate
ICS: inhaled corticosteroids
GPCR: G protein-coupled receptor
MCh: methacholine
NAM: negative allosteric modulator
PBS: phosphate-buffered saline
Penh: enhanced pause
PK/PD: pharmacokinetics/pharmacodynamics
PLA: poly-L-arginine
OVA: ovalbumin
RH: relative humidity
SEM: scanning electron microscopy
