# Supplementary material for: Characterisation of negative allosteric modulators of the calcium-sensing receptor, CaSR, for repurposing as a treatment for asthma
Source: J Pharmacol Exp Ther. Author manuscript; Available in PMC 2026 Oct 24. (PMC7619628; doi:10.1124/jpet.120.000281)
Supplement: Supplementary file 1 [file EMS217280-supplement-Supplementary_Material.pdf]

Supplementary Methods

Estimation of lung deposited dose, allometric scaling in humans, formulation and drug stability studies

These studies were performed by Cardiff Scintigraphics (Cardiff, United Kingdom). 1 mM aqueous alcoholic solutions of CaSR NAMs were established in propylene glycol followed by a 300-fold dilution into PBS for NPSP-795, Ronacaleret and JTT-305 (final drug concentrations 3 µM; final propylene glycol content 0.3%); AXT-914 was insoluble in propylene glycol, so it was dissolved in propylene glycol 0.27% + ethanol 0.03%. Because of the structural similarities between NPSP-795 and Ronacaleret, further analysis was carried out on only the amino alcohol NPSP-795 and the structurally unrelated quinazolin-2-one AXT-914.

The aerosol delivery measurements of NPSP-795 were carried out from a nebuliser formulation which was 100-fold higher in concentration than in the previous *in vivo* pharmacology studies (i.e., 300 µM). Approximately 2840 µg of NPSP-795 in 600 µl of DMSO were diluted to 20 ml of PBS (equivalent to approximately 142 µg/ml) and aerosol particles generated using a Philips Respironics Sidestream reusable nebuliser connected to a Philips Respironics Innospire Deluxe Compressor and to an exposure chamber (Philips Hospital & Health Care Amsterdam, Noord-Holland, NL). The Sidestream nebuliser was operated for 10 min, forming a steady-state aerosol cloud in the box. To determine the amounts delivered to the chamber and particle size distribution, after disconnecting and weighing the nebuliser a

Dosage Unit Sampling Apparatus (DUSA, Copley Scientific, Colwick, UK) tube was connected to a Next Generation Impactor^{TM} (NGI, MSP Corporation, USA) and operated with a negative flow rate of 28.3 L/min for 2 min to empty the exposure chamber. The process was repeated 8 times. The DUSA tube, filter and external filter were washed to recover the NPSP-795 for HPLC analysis (Agilent Technologies, Santa Clara, CA, USA). The average mass of solution aerosolized over the 10 min periods to generate data for NGI analysis was 1.12 g and the characteristics of the NPSP-795 aerosol are reported in Supplementary Table 2. To estimate the lung deposited dosages in the pharmacology studies from *in vitro* aerosol measurements using the Sidestream nebuliser and to provide an indication of the likely target lung dose in human studies we used the following equation:

LDD = C x IF x RMV x T

Where:

LDD = lung deposited dose of NPSP-795 (µg)

C = atmospheric concentration of NPSP-795 (µg/l)

IF = Inhaled fraction

RMV = respiratory minute volume (l/min)

T = time, i.e. the duration of exposure (min)

84.05 µg of NPSP-795 was collected from 8 evacuations of the exposure chamber following nebulisation of a 300 µM solution. Given the volume of the exposure chamber (15.2 l), the inhaled fraction (estimated from the fine particle fraction of which 86.59% of particles were <3µm in diameter), the respiratory minute volume of BALB/c mice (0.024 l/min, determined from a tidal volume of 0.15 ml and a respiratory rate of 160 breaths/min) and the duration of exposure used in the pharmacology studies (60 minutes), this equates to an atmospheric concentration of NPSP-795 of 0.69 µg/l. To correct for the 3 µM concentration used in the pharmacology studies, a value of 0.0069 µg/l is used in the equation. Using these values gives an estimated lung dosage of ~9 ng of NPSP-795 in a BALB/c mouse from nebulisation of a 3 µM solution over 60 min.

The estimated human dosage of NPSP-795 varies from 2 µg using allometric (body surface area) scaling, to 28 µg using theoretical scaled lung weight, 30 µg using scaled body weight and 58 µg using reported lung weights. From these estimates a value of ~30 µg was selected as a basis for the formulation stability studies.

Nebulizer solutions with a concentration of 0.4 mg/ml were prepared since only approximately 10% of the amount of drug in the nebuliser (i.e. 100 µg) is likely to be deposited in the lung in human studies. For solubility and stability testing, 2 mg of NPSP-795 were dissolved in either 100 µl of absolute ethanol (EtOH) or 150 µl of propylene glycol (PG) and aliquots kept in different storage conditions. Subsequently, one mL of 0.9 % w/v NaCl was added to the NPSP-795 in EtOH or PG and both formulations were subjected to 20 min centrifugation (SciQuip Ltd, SciSpin One Compact Centrifuge) at 2000 rpm before sampling the clear solutions. Approximately 1.5 ml of the EtOH and PG NPSP-795 in NaCl solutions were transferred into 3 separate Eppendorf tubes before storing at ambient room temperature, 5°C and at 40°C / 75 % relative humidity (RH). At each time point 25 µl of this sample were transferred to a 10 ml volumetric flask and diluted to volume with HPLC Recovery Solution (30% acetonitrile). 2 ml samples were transferred to HPLC vials for analysis.

The solubility of 400 µg/ml AXT9-14 in EtOH and PG were comparable and required high concentrations of co-solvent to ensure complete solubility, illustrating the poor solubility of AXT-914 in both aqueous EtOH and PG solutions. To ensure complete solubility of AXT-914 in aqueous EtOH solutions requires EtOH to be at an approximate minimum concentration of 53% v/v. For toxicology studies, this value would need to be increased to at least 60% v/v to reduce the potential for precipitation during nebulisation. However, as a nebuliser formulation this currently exceeds approved levels for human use. In aqueous PG solutions an approximate minimum concentration of 71% w/w was estimated to ensure complete solubility of AXT-914. For toxicology studies, the use of a 100% PG nebuliser formulation is likely to be needed, indicating that neither EtOH, nor PG solutions can be generated for human studies

with AXT-914. Therefore, it may be preferred to formulate AXT-914 as a dry power inhaler formulation.

AXT-914 micronization and characterization of the milled material

To test the hypothesis that AXT-914 can be micronized to particles of respirable size (around 3 µm), samples of AXT-914 (~1g) were milled by one pass through a jet-mill (Labo Mill Micronization Equipment FPS 0447, Italy) at 6 Bar injection pressure, and 7 Bar grinding pressure at controlled room humidity and temperature (50% RH, 20°C). After milling, samples were stored in desiccator for a minimum of 24 hours prior to analysis. Particle size distribution was assessed via dynamic laser diffraction (Malvern Mastersizer 3000, Malvern Instruments, Malvern, UK). The samples were dispersed in cyclohexane at 3500 rpm to reach obscuration between 0.1 and 20% (Hydro MV, Malvern Instruments, Malvern, UK). Particle size was recorded in a cycle of 5 measurements of 10 s. Briefly, samples were dispersed on a metal stub covered with adhesive carbon tape and sputter-coated with 15 nm gold (Sputter coater S150B, Edwards High Vacuum, Sussex, UK). A field-emission scanning electron microscope (SEM, Zeiss Ultra Plus, Carl Zeiss NTS GmbH, Germany) was used to assess particle size morphology at 500x magnification. Thermal responses of AXT-914 before and after the jet-milling process were measured using differential scanning calorimetry (DSC model 823e, Mettler Toledo International Inc., Schwerzenbach, Switzerland) at a heating rate of 10 °C/min between 25 and 400C, under an inert gas stream (N2, 25 cm^3/min). Samples (ca. 4-6 mg) were loaded and crimp-sealed in 40µl aluminium crucibles with pierced lids to ensure constant pressure. Melting temperature was determined from the maximum of the melting endotherm observed during the heating scan.

The response of milled AXT-914 to relative humidity was assessed using dynamic vapour sorption (DVS Intrinsic, Surface Measurement Systems Limited, London, UK). Approximately 7.5 mg of milled sample was loaded into the stainless-steel pan and dried at 0% RH prior to analysis. The samples were exposed to two 0-90% RH cycles at 25C with 10% RH increment.

Equilibrium of moisture was determined by a change in mass-to-time ratio (dm/dt) of 0.0005% min^{-1}. The data are presented as the sample mass change (%) over the investigated RH range.

Supplementary Figures

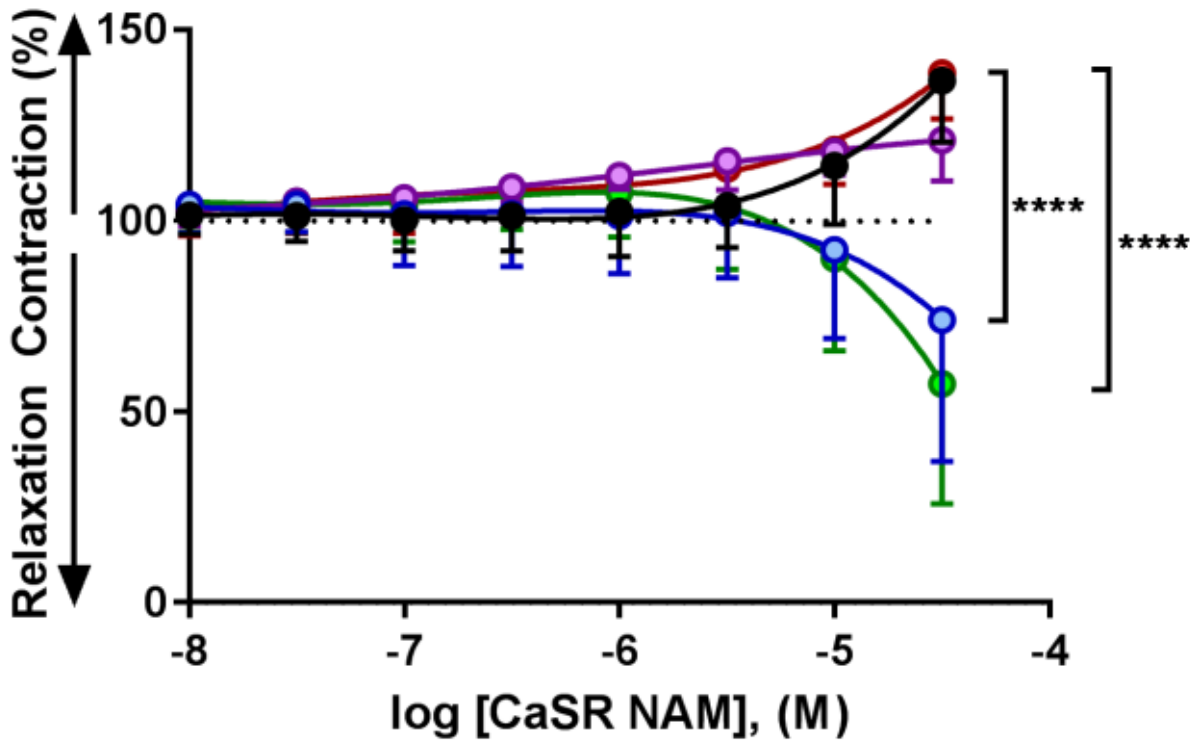

Supplementary Figure 1. Effects of amino alcohol and quinazolin-2-one CaSR NAMs on tracheal tone at physiological calcium concentrations.

Summary data showing effects of DMSO vehicle or CaSR NAMs on the ACh-induced tone in medium containing physiological free ionised calcium concentrations (1 mM Ca^{2+}). Data are shown as mean ± SD; ANOVA with Dunnett's *post hoc* test, **** p<0.0001; N = 4-6 trachea rings from individual animals per each experimental group.

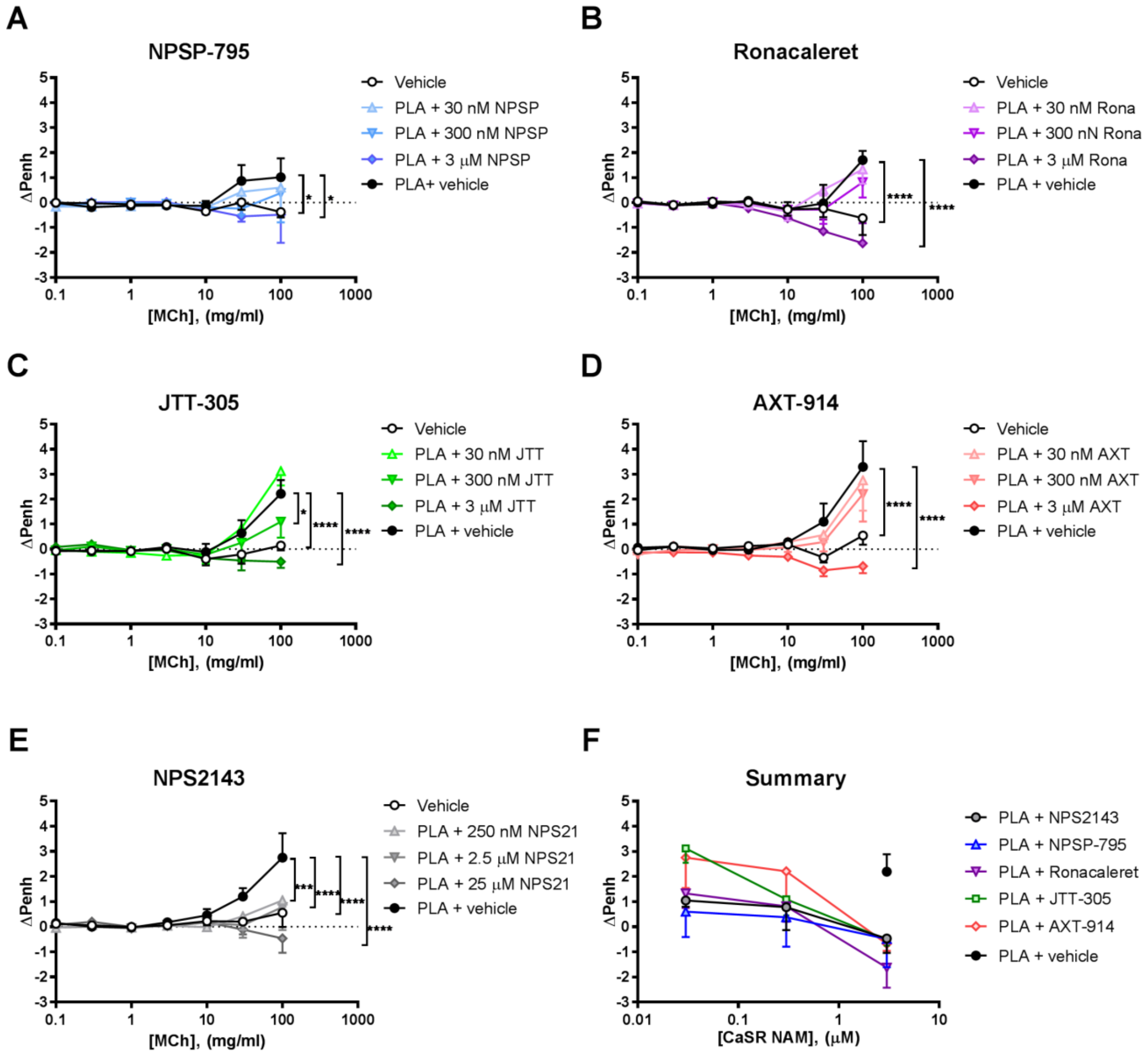

Supplementary Figure 2. Effects of increasing doses of inhaled CaSR NAMs on PLA-induced AHR *in vivo*.

A-E. PLA-induced airway obstruction (measured as ΔPenH) in mice challenged with methacholine (MCh, 0.1-100 mg/ml) after pre-treatment with different doses of CaSR NAMs previously tested in the clinic [NPSP-795 (**A**), Ronacaleret (**B**), JTT-305 (**C**), AXT-914 (**D**)] and of NPS2143 (**E**, positive control). **F.** Summary graph showing the effects of different doses of CaSR NAMs on PLA-induced airway obstruction in mice challenged with 100 mg/ml inhaled MCh. Data are shown as mean ± SEM, N = 6 animals per experimental group. Statistical comparisons: ANOVA with Dunnett's *post hoc* test, * p<0.05; ** p<0.01; *** p<0.001, **** p<0.0001.

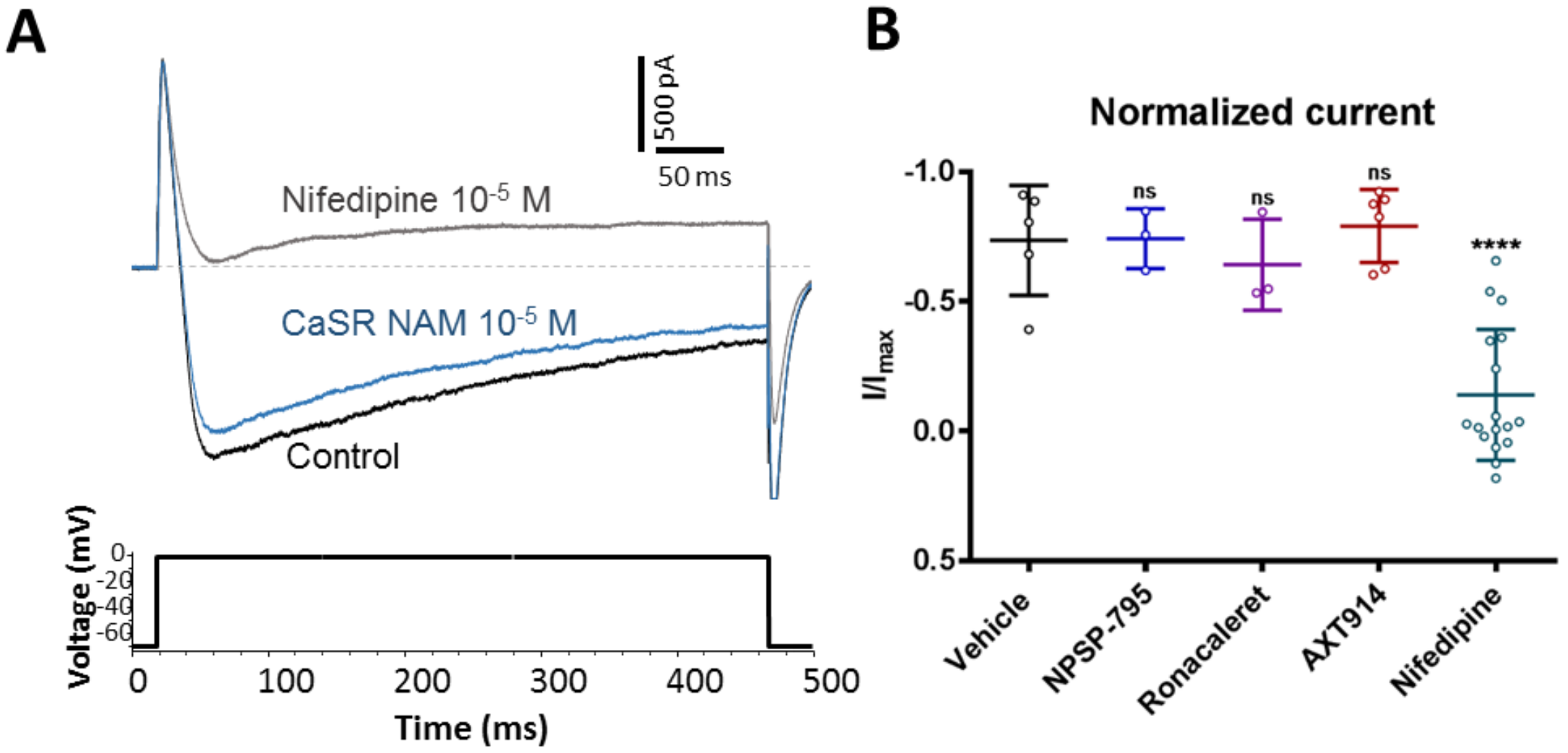

Supplementary Figure 3. Effect of CaSR NAMs on cardiac L_{Ca} channel currents.

A. Exemplar traces of Ba^{2+} current recorded in HEK293 cells expressing cardiac L_{Ca} channel α1c subunit (upper panel) using the voltage step protocol (bottom panel).

B. Effects of CaSR NAMs on normalized currents through L_{Ca}. Data are shown as scatter dot plot ± SD; ANOVA with Holm-Sidak's *post hoc* test, **** p<0.0001; N = 3-17.

A

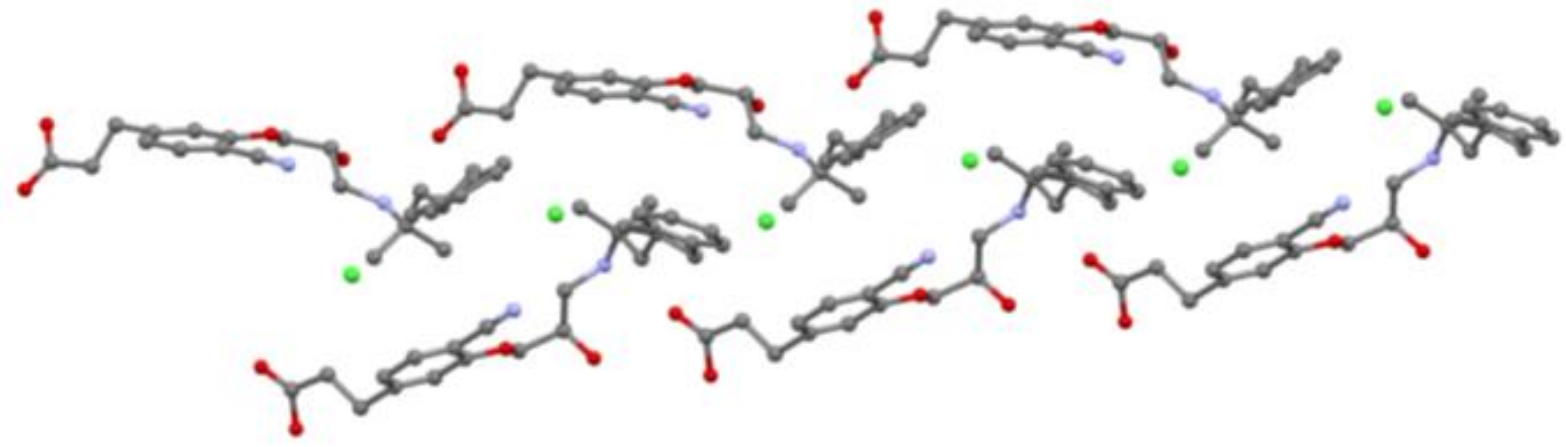

B

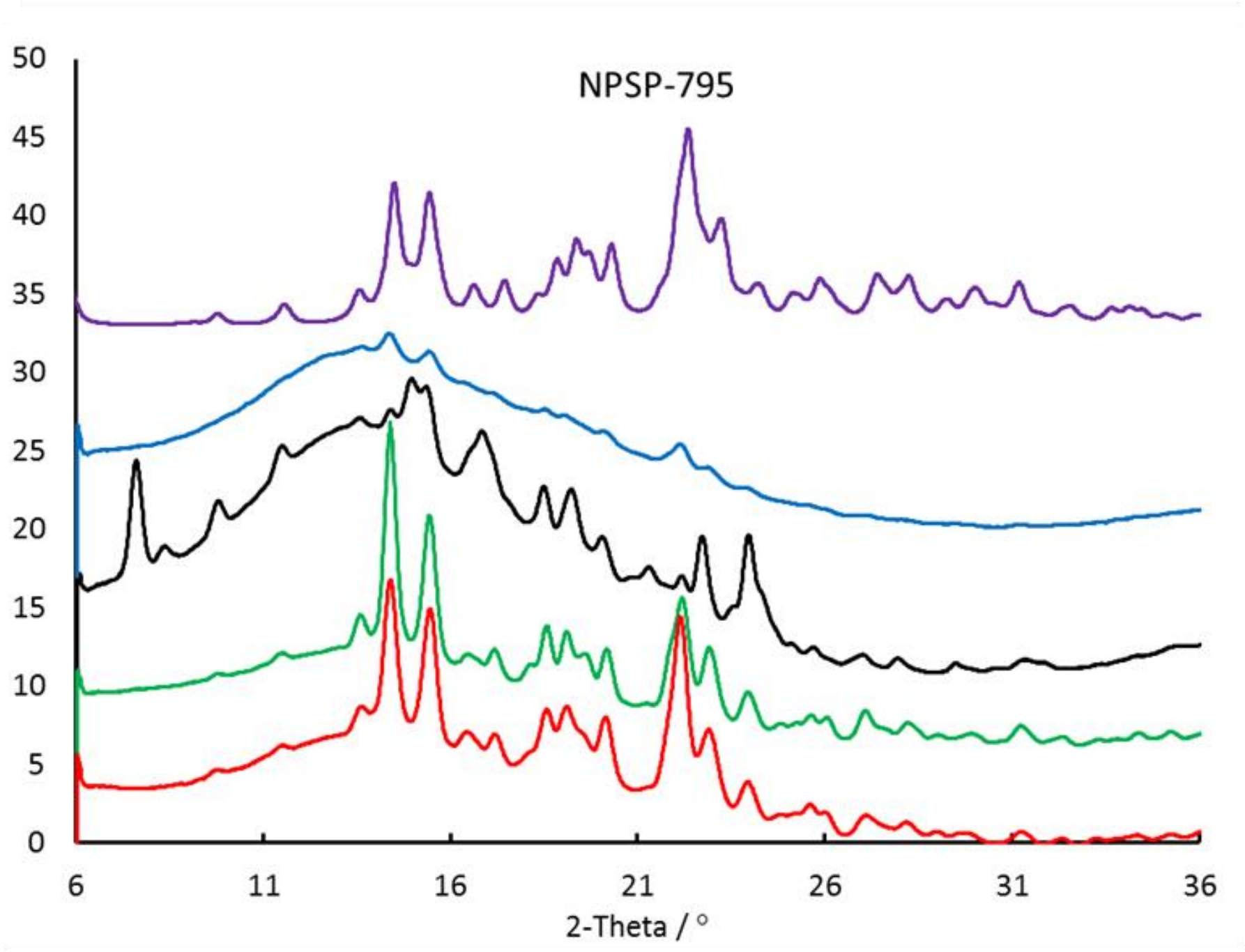

Supplementary Figure 4. Crystallography of NPS-P795
A. A segment of the structure of NPSP-795 that was crystallised by slow evaporation of acetone solvent and the crystal structure was determined by single crystal diffraction.
B. Powder diffraction shows the crystal structure of NPSP-795. The *purple* plot shows a powder pattern simulated from the single crystal data and is identical to the data for the original sample (*red*). The powder diffraction plot for the first crop on crystallisation from acetone (*green*). The final crop (obtained after further crystallisation of the material from acetone is shown in *black*. A sample of the original material ground in a mortar and pestle (*blue*).

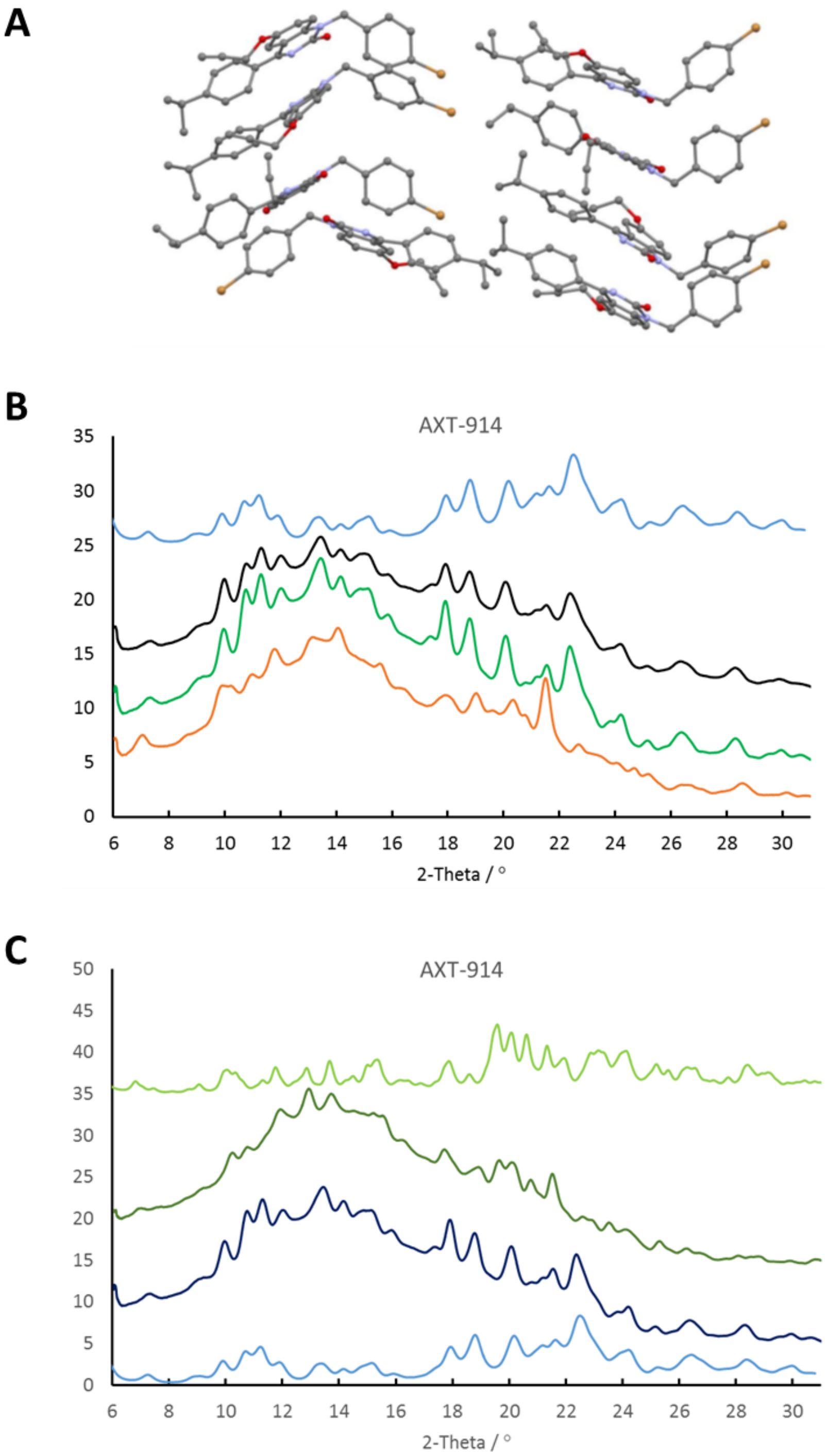

Supplementary Figure 5. Crystallography of AXT-914
A. A segment of the structure of AXT-914 that was crystallised by slow evaporation of acetone solvent and the crystal structure was determined by single crystal diffraction. **B.** Comparison of the powder x-ray diffraction pattern recorded before recrystallisation (orange), after recrystallization from acetone (green), after grinding with mortar and pestle to a fine powder (black), and the pattern simulated from the single crystal data (blue). **C.** Comparison of the powder x-ray diffraction pattern recorded after recrystallization from acetone (dark blue), ethanol (dark green), and the pattern simulated from the single crystal data for the acetone (light blue) and ethanol (light green) phases.

A

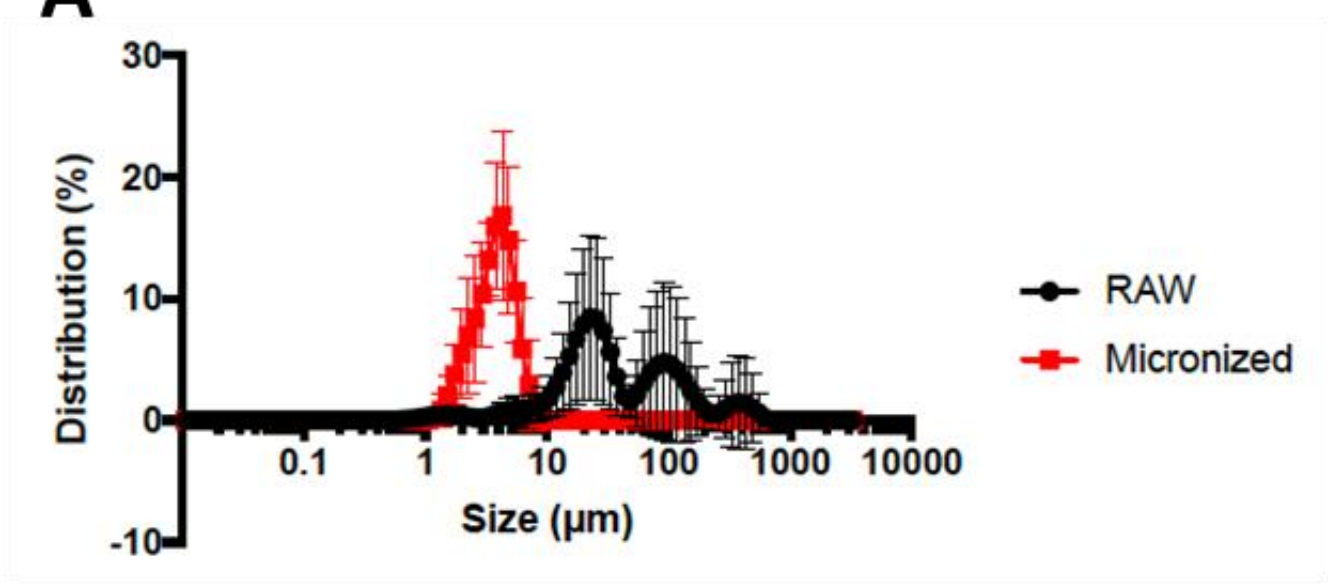

Result	RAW	Jet-milled
Span	4.465	1.222
Dx (10)	10.8 µm	1.73 µm
Dx (50)	33.9 µm	3.59 µm
Dx (90)	162 µm	6.11 µm

B

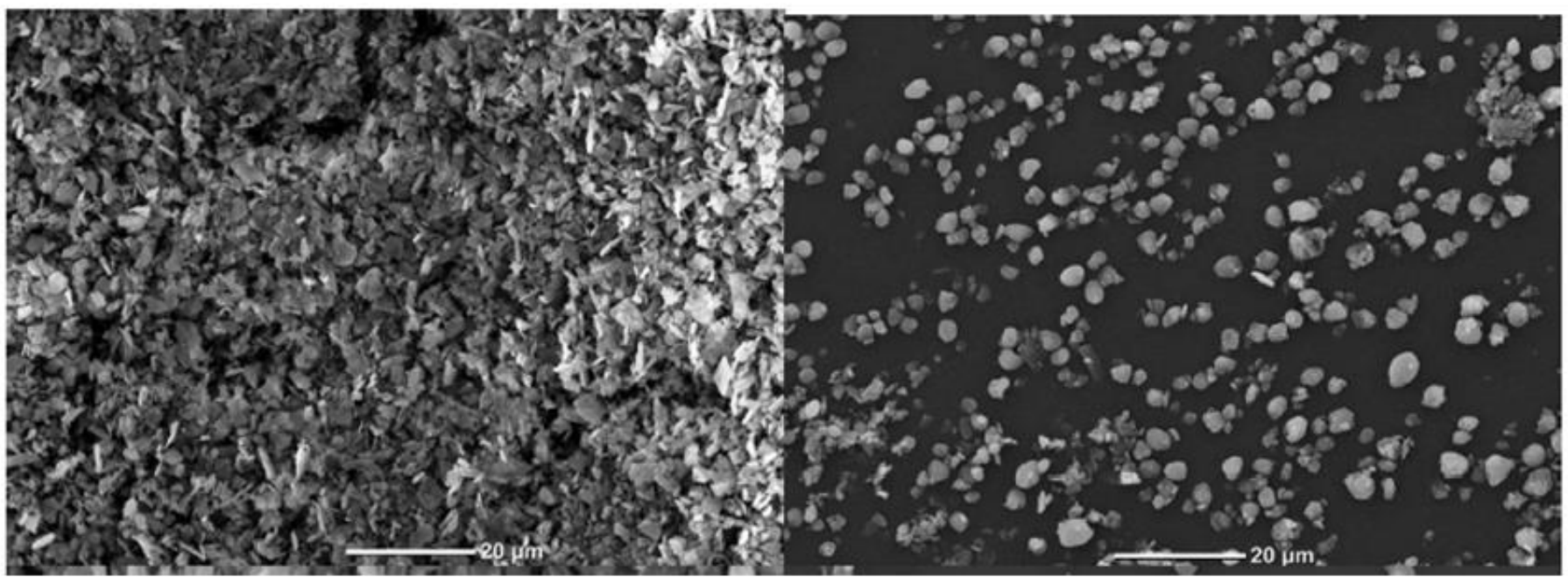

C

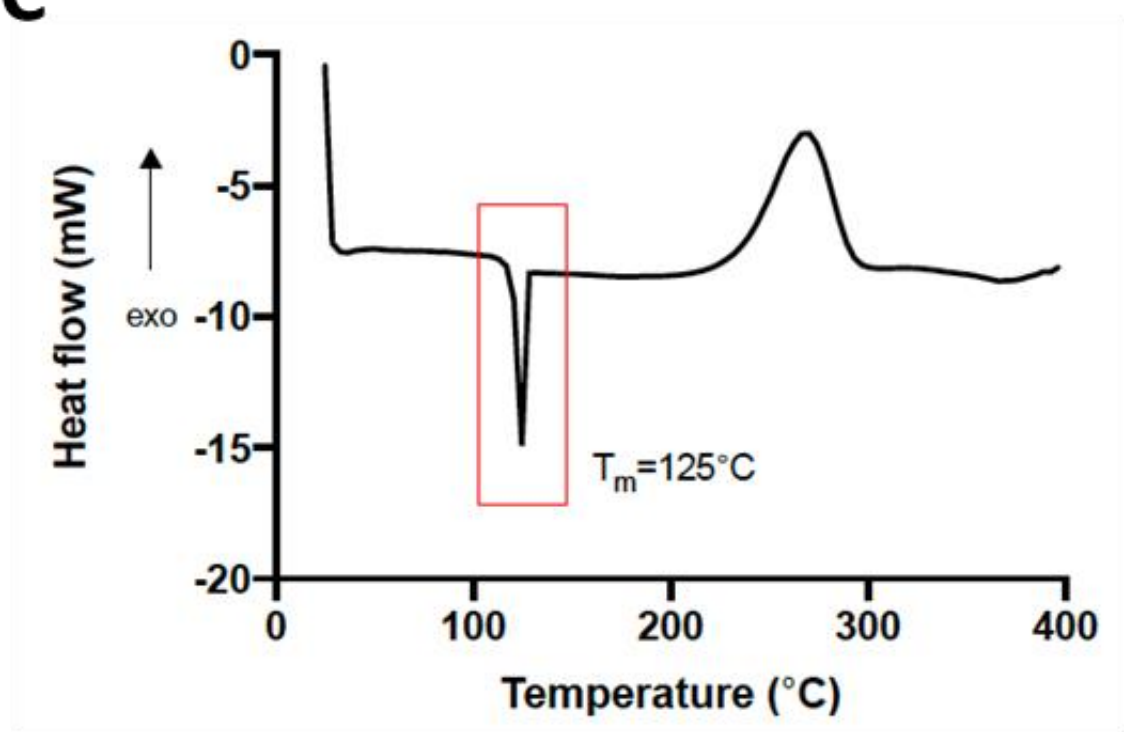

D

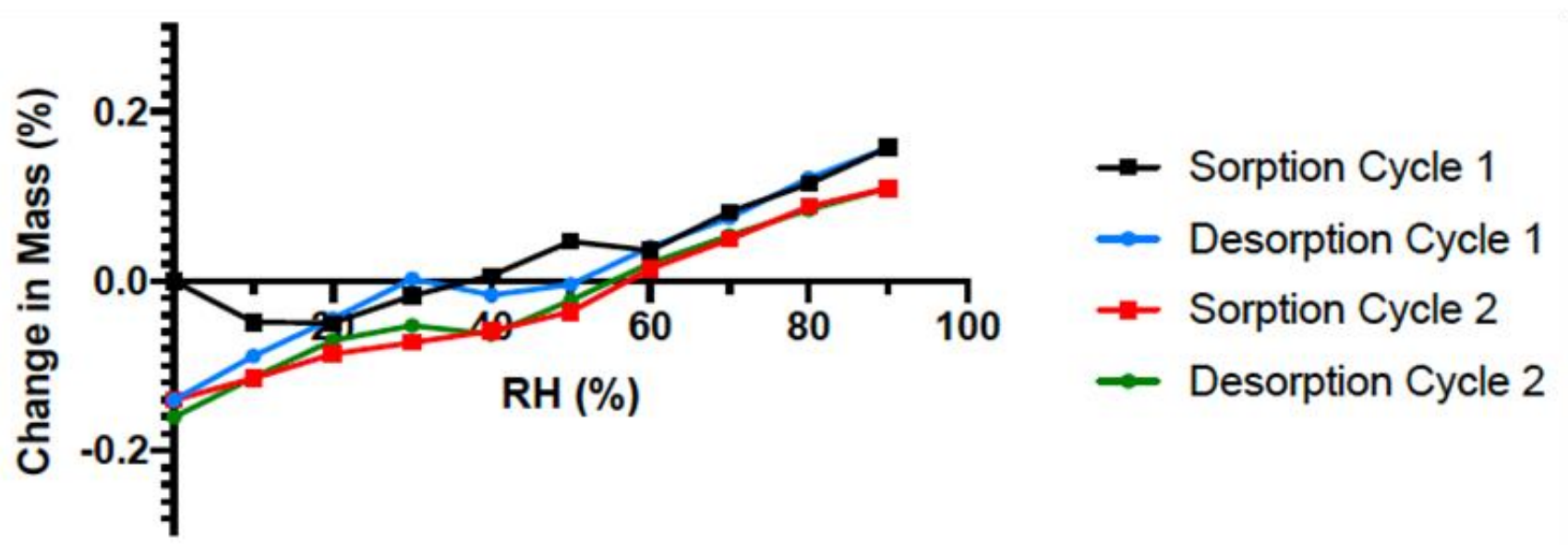

Supplementary Figure 6. AXT-914 can be micronized into particles of a respirable size range.

A. particle size distribution and size parameters of the raw and micronized AXT-914. **B**: SEM images of the raw and micronized AXT-914 (2000x). **C**. Thermogram of the milled AXT-914 showing an endothermic peak at 125ºC and an exothermic peak at 270ºC which may be related to the degradation of the substance. **D**. Vapor sorption shows minimal weight gain and hysteresis when the material is exposed to two cycles of 0-90% RH.

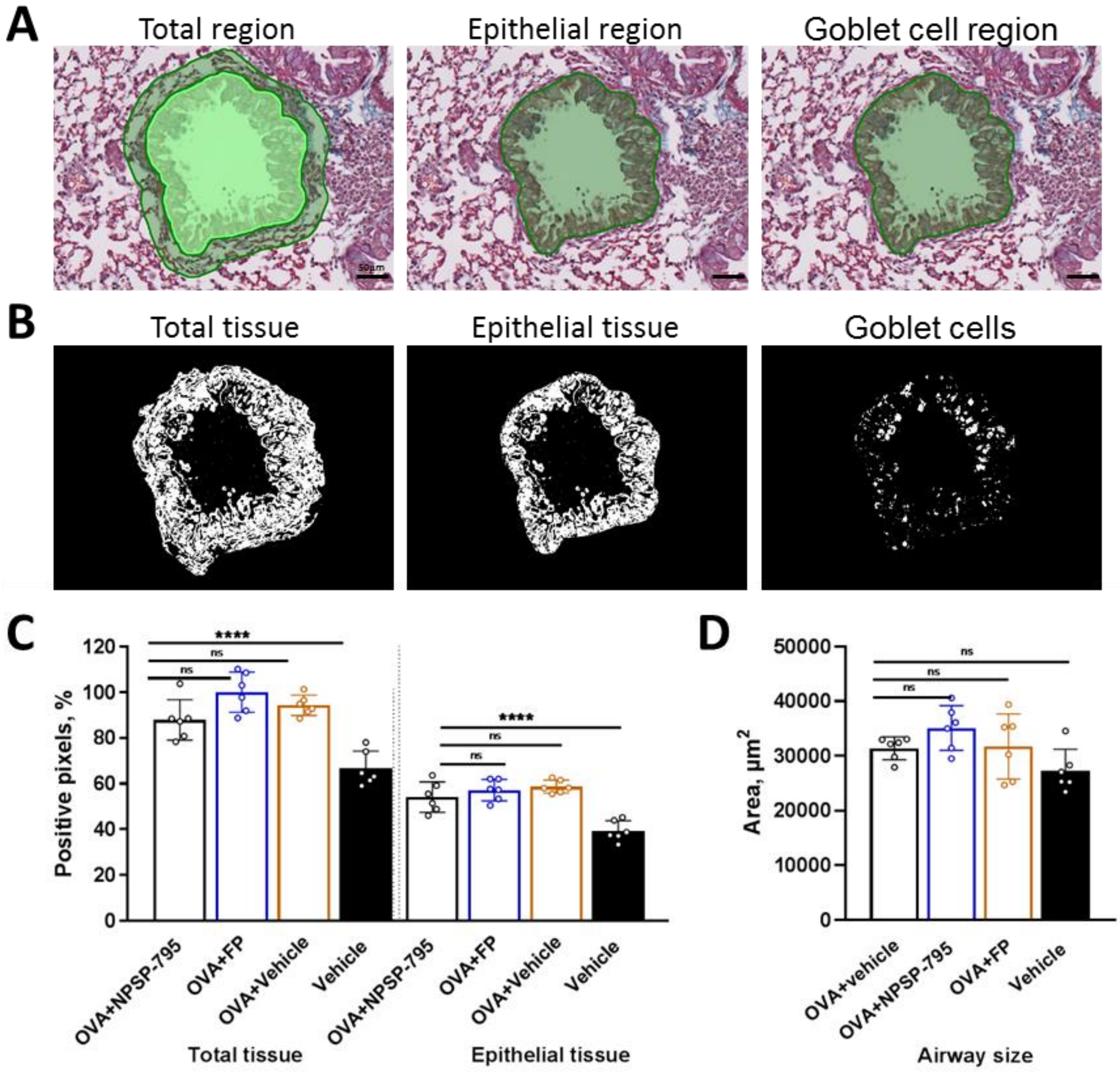

Supplementary Figure 7. Quantification of airway remodelling using StrataQuest image analysis software.

Manually identified regions of interest (**A**) were used to determine remodelling. Exclusion zones were set to eliminate interfering or unidentifiable objects such as cross section error. Red (cytoplasm), nuclei (brown), collagen (dark blue) and mucous (light blue) tissues were separated into grey layers based on colour using multiple reference shades for each colour. Positive signal was then “thresholded” to exclude weak positive staining and produce a mask image (**B-D**) that was used for subsequent area measurements. **B**: “Total tissue” was calculated as a % of the total region area (% of positive pixels). **C**: “Epithelial tissue” was calculated as a % of the epithelial region surface area. **D**: “Goblet cells” was calculated as a % of the goblet cell region. Balbc mice were sensitised and challenged with OVA as described in Figure 5 and the effects of inhaled NPSP-795 or inhaled FP on total tissue and epithelial remodelling (**C**) were quantified using StrataQuest image analysis software (TissueGnostics, AT). An average of 16 smaller airways of comparable size (**D**; <40,000 μm^2; n = 3-21) were taken from 3-4 Masson’s trichrome stained lung sections from each animal (N = 6 per condition) with the experimenter blinded to the condition using the opensource blindrename.pl script (Salter, 2016). Data are shown as scatter dot plot ± SD; ANOVA with Holm-Sidak’s multiple comparisons *post hoc* test; **** $p < 0.0001$.

Identification code	AXT-914-MeOH	NPSP-795	AXT-915-ACET
CCDC code	1915996	1915994	1915995
Empirical formula	$C_{27}H_{23}BrN_2O_2$	$C_{26}H_{33}ClN_2O_4$	$C_{27}H_{23}BrN_2O_2$
Formula weight	487.38	472.99	487.38
Temperature /K	150(2)	150(2)	150(2)
Wavelength / Å	1.54184	1.54184	1.54184
Crystal system	Monoclinic	Monoclinic	Monoclinic
Space group	$P2_1/c$	$P2_1$	$P2_1/c$
a / Å	19.3442(10)	13.0562(4)	16.485(2)
b /Å	34.3448(16)	12.5117(4)	35.013(5)
c / Å	17.3429(5)	15.2734(5)	16.813(3)
b /°	95.768(4)	92.742(3)	104.558(18)
Volume / Å3	11463.8(9)	2492.13(14)	9393(3)
Z	20	4	16
Density (calculated)/ Mg/m3	1.412	1.261	1.379
Absorption coefficient/ mm-1	2.651	1.631	2.589
Crystal size /mm3	0.351 x 0.033 x 0.026	0.192 x 0.132 x 0.088	0.332 x 0.040 x 0.026
Reflections collected	131119	20023	22298
Data / restraints / parameters	23082 / 30 / 1451	9698 / 1 / 603	4938 / 1248 / 1117
Goodness-of-fit on F2	1.025	0.983	1.025
R1 [I>2σ(I)]	0.0917	0.0580	0.0981
wR2 [I>2σ (I)]	0.2170	0.1493	0.2389
R1 (all data)	0.1658	0.0774	0.1925
wR2 (all data)	0.2728	0.1651	0.3156

Supplementary table 1. Crystal structure refinement data.

Parameter	Value
Average Rate of Aerosol Delivery (g/min)	0.11
FPF (% $<3\mu m$)	86.59
MMAD (μm)	1.43
GSD	2.19
FPD (μg <3 μm)	71.89

Supplementary table 2. Characteristics of NPSP-795 aerosol.
Abbreviations: FPF: fine particle fraction; MMAD: Mass median aerodynamic diameter; GSD: Geometric standard deviation; FPD: Fine particle fraction.
